# A New Target Organ of *Leishmania (Viannia) braziliensis* Chronic Infection: The Intestine

**DOI:** 10.3389/fcimb.2021.687499

**Published:** 2021-07-14

**Authors:** Amanda Gubert Alves dos Santos, Maria Gabriela Lima da Silva, Erick Lincoln Carneiro, Lainy Leiny de Lima, Andrea Claudia Bekner Silva Fernandes, Thaís Gomes Verzignassi Silveira, Debora de Mello Gonçales Sant’Ana, Gessilda de Alcantara Nogueira-Melo

**Affiliations:** ^1^ Biosciences and Physiopathology Program, Universidade Estadual de Maringá, Maringá, Brazil; ^2^ Department of Clinical Analysis and Biomedicine, Universidade Estadual de Maringá, Maringá, Brazil; ^3^ Department of Morphological Sciences, Universidade Estadual de Maringá, Maringá, Brazil

**Keywords:** leishmaniasis, small intestine, inflammation, enteric nervous system, TGF-beta

## Abstract

*Leishmania (Viannia) braziliensis* is one of the main causes of cutaneous leishmaniasis in the Americas. This species presents genetic polymorphism that can cause destructive lesions in oral, nasal, and oropharyngeal tracts. In a previous study, the parasite caused several histopathological changes to hamster ileums. Our study evaluates immune response components, morphological changes, and effects on neurons in the ileums of hamsters infected by three different strains of *L. (V.) braziliensis* in two infection periods. For the experiment, we separated hamsters into four groups: a control group and three infected groups. Infected hamsters were euthanized 90- or 120-days post infection. We used three strains of *L. (V.) braziliensis*: the reference MHOM/BR/1975/M2903 and two strains isolated from patients who had different responses to Glucantime^®^ treatment (MHOM/BR/2003/2314 and MHOM/BR/2000/1655). After laparotomy, ileums were collected for histological processing, biochemical analysis, and evaluation of neurons in the myenteric and submucosal plexuses of the enteric nervous system (ENS). The results demonstrated the increase of blood leukocytes after the infection. Optical microscopy analysis showed histopathological changes with inflammatory infiltrates, edemas, ganglionitis, and *Leishmania* amastigotes in the ileums of infected hamsters. We observed changes in the organ histoarchitecture of infected hamsters when compared to control groups, such as thicker muscular and submucosa layers, deeper and wider crypts, and taller and broader villi. The number of intraepithelial lymphocytes and TGF-β-immunoreactive cells increased in all infected groups when compared to the control groups. Mast cells increased with longer infection periods. The infection also caused remodeling of intestinal collagen and morphometry of myenteric and submucosal plexus neurons; but this effect was dependent on infection duration. Our results show that *L. (V.) braziliensis* infection caused time-dependent alterations in hamster ileums. This was demonstrated by the reduction of inflammatory cells and the increase of tissue regeneration factors at 120 days of infection. The infected groups demonstrated different profiles in organ histoarchitecture, migration of immune cells, and morphometry of ENS neurons. These findings suggest that the small intestine (or at least the ileum) is a target organ for *L. (V.) braziliensis* infection, as the infection caused changes that were dependent on duration and strain.

## Introduction

*Leishmania (Viannia) braziliensis* is one of the main species that causes cutaneous and mucocutaneous leishmaniasis in the Americas (Patino et al., 2020). In 2018, the World Health Organization reported 253,435 new cases of cutaneous leishmaniasis. More than 46,000 of these cases were in the Americas (World Health Organization, 2020); 84% of these cases occurred in Brazil (Pan American Health Organization, 2019), that is considered a “high burden country” for leishmaniasis (World Health Organization, 2020). The disease primarily affects poor segments of the population and results in negative social and economic impacts (Ministério da Saúde, 2017).

The diversity of clinical forms of leishmaniasis caused by *L. (V.) braziliensis* and disease severity are related to the immune system (Fernandes et al., 2016), genetic factors, the clinical condition of the host (Quaresma et al., 2018), and the inoculum (Ribeiro-Romão et al., 2014) and strain of the parasite (Vieira et al., 2019); this species presents high levels of genetic polymorphism (Patino et al., 2020). Different strains found in the same region can cause different clinical forms (Guimarães et al., 2016; Quaresma et al., 2018; Rugani et al., 2018) and therapeutic responses (Fernandes et al., 2016; Gagini et al., 2017), even in the same patient (Hoyos et al., 2019). Lesions are destructive and principally affect the skin and mucus membranes in the oral, nasal, and oropharyngeal tracts (Ministério da Saúde, 2017; Conceição-Silva and Morgado, 2019). Furthermore, studies have reported the occurrence of lesions in the eye (Cruz et al., 2017) and larynx (Silva et al., 2017). The DNA of the parasite has also been detected in bone marrow of immunocompromised patients (Gontijo et al., 2002; Silva et al., 2002).

Studies have reported the presence of amastigotes of the parasite in spleens, lymph nodes (Almeida et al., 1996; Gomes-Silva et al., 2013; Ribeiro-Romão et al., 2014), and livers (Barral et al., 1996) of animals infected with *L. (V.) braziliensis.* In chronically-infected hamsters, our research group detected the DNA of the parasite in ileums and mesenteric lymph nodes, amastigotes in the ileums, and alterations to intestinal architecture (Santos et al., 2018b). Hamsters are good models for *L. (V.) braziliensis* infection research, as they develop lesions (De Oliveira et al., 2004; Gomes-Silva et al., 2013), clinical and histopathological manifestations (Almeida et al., 1996; Gomes-Silva et al., 2013), and immune responses (Ribeiro-Romão et al., 2014) that are similar to those observed in human leishmaniasis. With hamsters, the parasites can migrate to the viscera or other skin sites, where the parasites replicate (Almeida et al., 1996).

The effect of *Leishmania* infection in the gastrointestinal tract is the topic of studies that evaluated different segments of the intestine of dogs (Figueiredo et al., 2014; Silva et al., 2016; Silva et al., 2018) and rodents (Souza et al., 2019; Lewis et al., 2020; Passos et al., 2020) with visceral leishmaniasis (VL). In human VL, the presence of amastigote forms in intestinal tissues may be related to episodes of diarrhea (Baba et al., 2006; Soria López et al., 2016; Raina et al., 2017) or not (Chattopadhyay et al., 2020). The findings have reported higher villi and moderate inflammatory infiltrate composed mainly of mononuclear cells in the lamina propria of the duodenum (Chattopadhyay et al., 2020) and mild ulcers in the colon (Baba et al., 2006).

The intestine is the largest immune organ in mammals; it helps to maintain the equilibrium of the organism (Chassaing et al., 2014). The ileum is the final segment of the small intestine and plays an important role in its immunity. The ileum has more Peyer patches (Mowat and Agace, 2014), the highest concentration of Paneth cells (Santaolalla et al., 2011; Mowat and Agace, 2014), lymphoid aggregates (Mowat and Agace, 2014), which may be related to the highest number of bacteria in this portion of the intestine (Santaolalla et al., 2011). Specialized cells (e.g., enterocytes, goblet cells, Paneth cells, and enteroendocrines) in its epithelium participate in the innate immune response. These cells secrete substances with various functions, such as glycoproteins, antimicrobial substances, cytokines, and hormones. The lamina propria has a high quantity and variety of immune cells, such as mast cells, macrophages, dendritic cells, and lymphocytes, among others (Abbas et al., 2017). Epithelial cells and the innate and adaptive immune systems interact with the ENS to promote immune tolerance, defense, and organ regeneration (Jacobson et al., 2021).

The ENS sends and receives nerve impulses from other organs and is responsible for digestion, motility (Yoo and Mazmanian, 2017), and maintenance of intestinal homeostasis (Drokhlyansky et al., 2020). Studies have shown that the intestine is affected by *L. (V.) braziliensis* infection (Santos et al., 2018a; Santos et al., 2018b) thus, evaluating the effects of the infection from different parasite strains on histoarchitecture and immune response of the organ and ENS are essential for the understanding of the complex *Leishmania-*host relationship. As in previous studies we detected changes in the ileum (Santos et al., 2018a; Santos et al., 2018b), we carried out this research to confirm that the intestine is a target organ for infection by *L. (V.) braziliensis*. Thus, the objective of this work was to evaluate some components of the immune response, morphological and neuronal alterations in this organ of hamsters infected by other three different strains of the parasite at two different periods.

## Materials and Methods

### Ethics Statement

The animal studies were previously approved by the Ethical Committee on Animal Use of the Universidade Estadual de Maringá (UEM) under protocol number 7587260416.

### Parasites

For the infection, we used three different strains of *L. (V.) braziliensis*: The World Health Organization reference strain MHOM/BR/1975/M2903 (2903) and two strains isolated from patients treated at the Laboratório de Ensino e Pesquisa em Análises Clínicas (LEPAC/UEM). The two strains from UEM came from patients who had different responses to Glucantime^®^ treatment. The patient who was infected with the MHOM/BR/2003/2314 (2314) strain showed good therapeutic response with complete lesion regression after the first treatment. The MHOM/BR/2000/1655 (1655) strain was isolated from a patient whose infection was considered resistant by reactivation of the previously-healed lesion. These isolates were cultured, cryopreserved, and identified by the Oswaldo Cruz Institute, Rio de Janeiro, Brazil (Fernandes et al., 2016).

### Experimental Design and Infection

For the infection, we used promastigotes in the stationary growth phase from the fifth *in vitro* passage. The parasites were cryopreserved in the Leishmaniasis Laboratory of UEM. For culture they were thawed and reactivated. They were then kept in a culture of 199 medium (Gibco Laboratories^®^, Grand Island, USA) and supplemented with 1% human urine, 10% fetal bovine serum, and 1% L-glutamine. For infection preparation, the hamsters were anesthetized with a combination of ketamine (Francotar^®^-Virbac Animal health) and xylazine (Calmiun Agener-Union Animal Health).

We used 48 female hamsters (*Mesocricetus auratus*) (21-day-old). The hamsters were randomly separated into four groups (n = 12/group): the control group and three groups inoculated with different isolates of *L. (V.) braziliensis.* The control group received an intradermal injection of 100 µl of phosphate-buffered saline (PBS) in the left hindpaw. The infected group received an intradermal injection of each isolate (2x10^7^/100 µl) in the left hindpaw. Once a week, both paws were measured using a digital pachymeter and analyzed for edema and lesions. The hamsters were weighed before infection and before euthanasia.

The hamsters were kept in a temperature-controlled environment with a light/dark cycle (12/12 hr). To avoid external contamination, we housed the animals in individually ventilated cages with autoclaved wood shavings and filtered air and water. Food and water were available *ad libitum*. The hamsters were euthanized 90- or 120-days post infection, thus forming a total of eight groups. For all experiments, we used 4–6 animals per group.

### Euthanasia and Tissue Collection

Before euthanasia, blood samples were collected from the retro orbital sinus and total leukocytes were counted using a Neubauer chamber. The differential leukocyte count was determined in blood smears (May-Grünwald-Giemsa staining technique) using light microscopy. The data is represented in box plots (median with 25 to 75 percentile), whiskers (2.5 to 97.5 percentile), and mean (+).

The hamsters were euthanized under deep anesthesia. We then performed the laparotomies and collected and measured the ileums. Approximately 1 cm of the ileum was collected for histology. The ileum samples were fixed in buffered paraformaldehyde, dehydrated, diaphanized, and embedded in paraffin. One segment (0.5 cm) was used for biochemical analyses; this fragment was washed with PBS, frozen in liquid nitrogen, and stored in a freezer at -80°C. A different segment (2 cm) was used for the evaluation of enteric neurons. This segment was fixed in 4% paraformaldehyde and immersed in the same fixative solution for 3 hours at room temperature. It was then opened along the mesenteric border, washed twice for 10 minutes with PBS, and stored in PBS with 0.08% sodium azide at 4°C.

### Histological Processing and Immunohistochemistry

To evaluate ileum morphology and cellularity, sets with semi-serial 5 µm transverse histological sections were prepared and stained using different techniques. Histopathological evaluations and morphometric analyses of ileal walls, enterocytes, and intraepithelial lymphocytes (IELs) were performed on sets stained with hematoxylin and eosin (HE) (Santos et al., 2018b; Passos et al., 2020). Goblet cells producing different mucins were counted in Alcian blue pH 1.0 (AB 1.0), Alcian blue pH 2.5 (AB 2.5), and periodic acid-Schiff (PAS) stained sets (1; Santos et al., 2018b). Total mast cells were counted with the toluidine blue technique (Yu et al., 2016; Pastre et al., 2019), and collagen fibers were analyzed in picrosirius red (Pastre et al., 2019; Panza et al., 2021).

The immunohistochemistry technique was used to label TGF-β and *Leishmania* amastigotes, as described by Santos et al. (2018b). Briefly, the slides were separately exposed to primary anti-*Leishmania* (1:200 dilution) produced in infected *L. (L.) amazonensis* mice and purified with intestines of healthy hamsters (Santos et al., 2018b) and anti-TGF-β (1:100 dilution; Thermo Fisher Scientific, Rockford, IL, EUA) antibodies. After incubation with the primary antibody, the sets were incubated with horseradish peroxidase polymer conjugate (Life Technologies Corp., Frederick, MD, USA) and stable DAB (Invitrogen™, Carlsbad, CA, USA), counterstained with Mayer’s hematoxylin, and mounted with coverslips. A brown color in the sets indicated a positive reading.

### Morphometric Analyses

**Figure 1:**
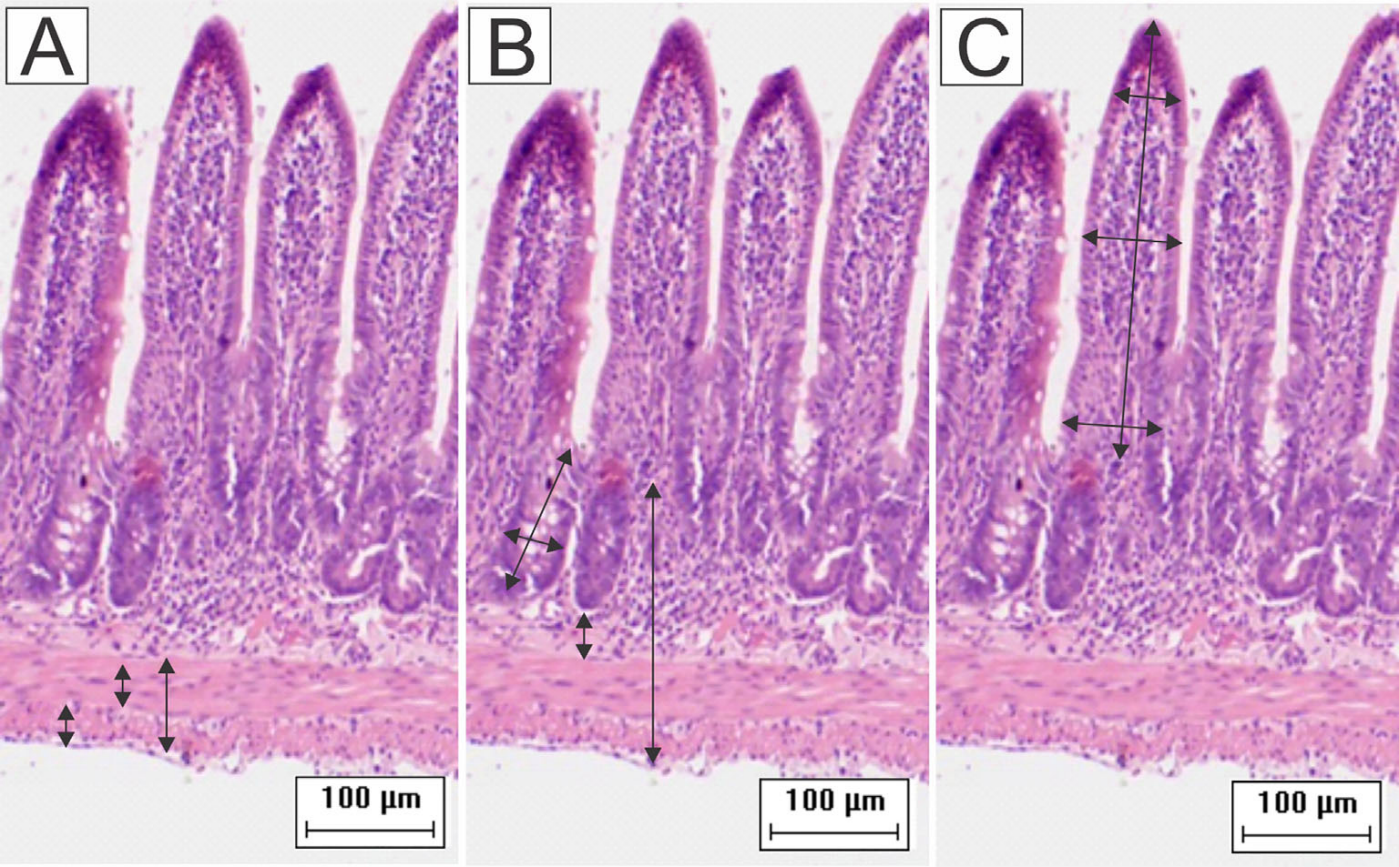
Schematic representation of the measurement of **(A)** muscular layers, **(B)** submucosa, total wall, depth and width of the crypts, and **(C)** height and width of the villi. Were performed 64 measurements of each parameter for each animal. To obtain the width of the villi, the base, middle, and apex were measured and then the average of these values resulted in the final value (HE staining, 20× magnification, scale bar = 100 µm, Olympus CX31).

Motic Images Plus (version 2.0) software was used to measure ileal walls and enterocytes. For these analyses, images were captured with a digital camera (Moticam 2000, 2.0 Megapixel) coupled to an optical microscope (MOTIC B5). Morphometries of ileal walls were performed in 16 images captured with a 10x objective lens. We performed 64 measurements of each of the following parameters for each animal: total thickness of intestinal walls, muscular tunics, and submucosa; widths and depths of crypts (**Figure 1**). The heights and widths of villi were measured using the same images. The base, middle, and apex of villi were measured first, and then the average of these values resulted in the final value (Santos et al., 2018b; Passos et al., 2020). For each animal, the heights and widths of 80 enterocytes and their respective nuclei were measured in images captured with a 100x objective lens (Santos et al., 2018b).

Image-Pro^®^ Plus (version 4.5.0.29) software was used for the evaluation of collagen fibers and HuC/HuD immunostaining neurons. In the slides stained with picrosirius red, 16 images were captured with a 20x objective lens in a light microscope (Olympus BX50 - Minato-Ku, Japan) with the use of a polarizing filter (Olympus U-POT, Japan) for measurement of type I and III collagen fibers. We captured 16 images without the polarizer for the measurement of total fibrillar collagen (µm²) (Pastre et al., 2019; Panza et al., 2021).

### Cell Counting

For the quantification of goblet cells, IELs, and mast cells, we used a Nikon Eclipse E200 optical microscope. Using the 40x objective lens, we counted the number of IELs and goblet cells in 2,560 epithelial cells of each hamster (160 epithelial cells/quadrant/cut). For statistical analysis, we calculated the ratio to 100 epithelial cells (Santos et al., 2018b). Total mast cells were counted in 100 microscopic fields using the 100x objective lens (Pastre et al., 2019). From ileal mucosa and submucosa, TGF-β-immunoreactive cells (TGF-β-IR) were counted in 16 images captured by an Olympus CX31 microscope attached to a digital camera (Moticam 2000, 2.0 Megapixel). Quantities of mast cells and TGF-β-IR cells in 1 mm^2^ were calculated.

### *Leishmania* Amastigote Analysis

From each hamster, four histological sections stained with HE were analyzed to verify amastigote forms. The positive hamsters had their histological slides submitted to immunohistochemistry with anti-*Leishmania* antibodies. Five immunostained tissue sections were examined for the presence of extra- or intramacrophagic amastigote forms. Positive (sections of popliteal lymph nodes) and negative controls (sections without the primary antibody) were used.

### Biochemical Analyses

Fragments of the ileum were homogenized with PBS (4mM) and centrifuged. The supernatant was then used for the measurement of nitric oxide (NO) and evaluation of enzyme myeloperoxidase (MPO) activity. The pellet was resuspended in PBS with hexadecyltrimethylammonium bromide (8mM), homogenized, and centrifuged to measure the enzymatic activity of N-acetyl-β-D-glucosaminidase (NAG). All analyses were performed in duplicate in a 96-well microplate, and their absorbances were measured in a microplate reader (Spectra Max Plus).

For the measurement of MPO, 10 μL of the sample reacted with the o-dianisidine solution (16.7 mg O-dianisidine dihydrochloride, 90 ml double-distilled water, 10 ml PBS, and 50 μl of 1% H_2_O_2_) for 5 minutes with protection from light. The enzymatic reaction was stopped by the addition of acetate solution, and the reading was performed at 450 nm. The results were expressed in optical density (OD).

The estimation of NO was performed indirectly by the determination of nitrite (NO^2-^) with the Griess method. 50 μL of the sample was incubated with Griess solution (1% sulfanilamide in 5% phosphoric acid, and 0.1% N-1-naphthylethylenediamine dihydrochloride in water) at room temperature. The NO concentration was calculated based on the sodium nitrite standard curve. The absorbance was measured at 550 nm; the results were expressed in μM concentration of NO^2−^.

Finally, the measurement of NAG enzymatic activity was performed with a 25 μL sample that remained incubated for 1 hour at 37°C with a citrate buffer and NAG solution (1.14 mg of p-nitrophenyl-N-acetyl-β-D- glucosamine in distilled water). Before the reading at 405 nm, glycine buffer was added. The result was expressed in OD/g of wet tissue.

### Neuron Counting and Morphometry

Small fractions of ileums were dissected under stereomicroscopy to obtain whole mount preparations of enteric plexuses. To mark the total population of HuC/HuD neurons, whole mounts of myenteric and submucosal plexuses were washed 3 times for 5 minutes with PBS (0.1M pH 7.4) and incubated separately in microtubes with an antigen blocking solution containing 3% bovine serum albumin (BSA; Sigma, St. Louis, MO, USA) and 0.1% Triton X100 (Sigma, St. Louis, MO, USA) diluted in PBS for 1 hour at room temperature. After this, the membranes were incubated in a solution containing the primary mouse anti-HuC/HuD (1:300; Molecular Probes, Eugene, OR, USA) antibody, 3% BSA, and 0.1% Triton X100 diluted in PBS for 48 hours under stirring at room temperature. After this period, the samples were washed 3 times for 5 minutes with PBS and incubated in a solution containing the secondary Alexa Fluor 488 donkey anti-mouse antibody (1:300; Molecular Probes, Eugene, OR, USA), 3% BSA, and 0.1% Triton X100 diluted in PBS for 2 hours at room temperature under agitation and protection from light. Then, the membrane preparations were washed 3 times for 5 minutes in PBS, mounted on glass slides with Prolong Gold Antifade (Molecular Probes, Eugene, OR, USA), and stored at 4°C (light-protected).

The counting of HuC/HuD-immunoreactive neurons from myenteric and submucosal plexuses was performed on 32 images captured randomly with a 20x objective lens in all areas of the ileum circumference using the FSXBSW Image Browser integrated in an Olympus FSX100 light microscope (Olympus, Tokyo, Japan) with immunofluorescence filters. In the submucosal plexus, we counted the neurons inside and outside the ganglia and the total number of ganglias. These results were expressed in cells/mm². The areas of 100 neurons (µm^2^) of submucosal and myenteric neurons per animal were measured in the same images. For both analyses, we used Image-Pro^®^ Plus (version 4.5.0.29) software.

### Statistical Analysis

The statistical analyses were performed using the data of the individual animals and were determined based on the data distribution, which was verified using the Shapiro-Wilk or D’Agostino Pearson tests (BioEstat 5.3 software). Comparisons between the groups were verified with two-way analysis of variance (ANOVA) followed by Tukey’s multiple-comparison test or Fisher’s *post hoc* test; the Kruskal-Wallis test followed by Dunn’s pos hoc test was also used (GraphPad Prism 8.0.1 software). We compared the control groups to the infected groups in the two experimental periods (90 or 120 days) and the 90-day infected group to the 120-day group. Values of *p <* 0.05 were considered statistically significant.

## Results

### Clinical Signs

**Figure 2:**
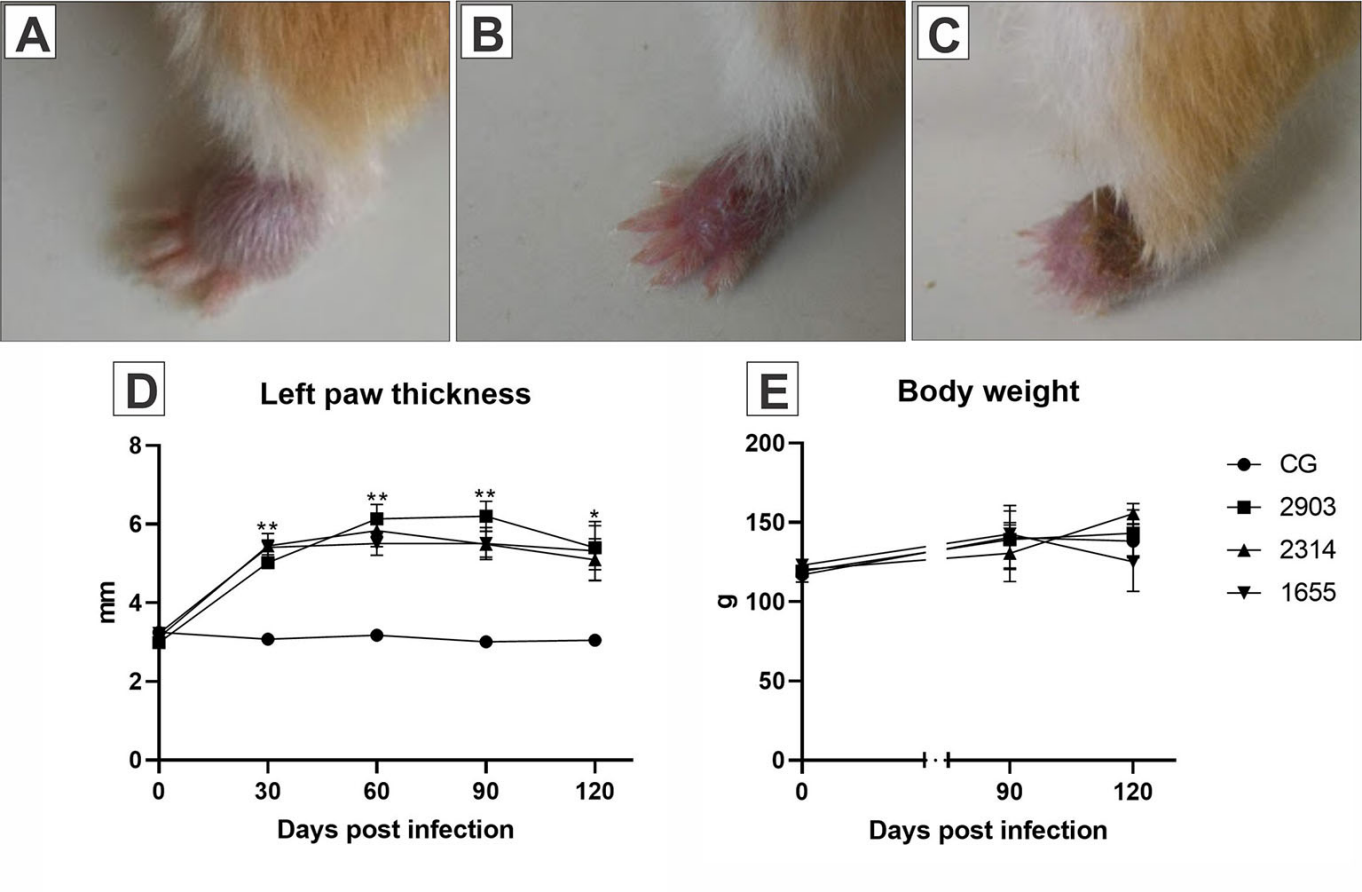
Images **(A–C)** represent the evolution of the paw lesion after the infection by *L.* (*V.) braziliensis* (MHOM/BR/2000/1655 strain): **(A)** initial edema, **(B)** initial lesion, **(C)** ulcerated lesion. **(D)** Represents the evaluation of the clinical course of lesions through the 120 days following the infections. The size of the lesions was measured with a digital pachymeter by evaluating the dorsal-ventral thickness of the infected paw (mm). *p < 0.05; **p < 0.001 when compared the infected groups to the respective control. **(E)** Demonstrates the body weight (g) of the hamsters before parasite inoculation and at 90- and 120-days post infection (*n* = 4). CG, control group. 2903: group infected with MHOM/BR/1975/M2903. 2314: group infected with MHOM/BR/2003/2314. 1655: group infected with MHOM/BR/2000/1655.

The infection was confirmed by the development of the lesion at the site of inoculation of the parasite, therefore, only animals with lesions on the left hindpaw were used. Body weight, consistency of feces, and appearance of hair did not change during the experimental period when compared the groups. Edema was observed in infected paws in the first days after infection. The lesions started to appear between the third and fourth week after infection and no statistical differences were observed among the infected groups. All infected hamsters showed difficulty in mobility due to lesion progression (**Figure 2**).

### Infection Increased Leukocytes in Peripheral Blood

**Figure 3:**
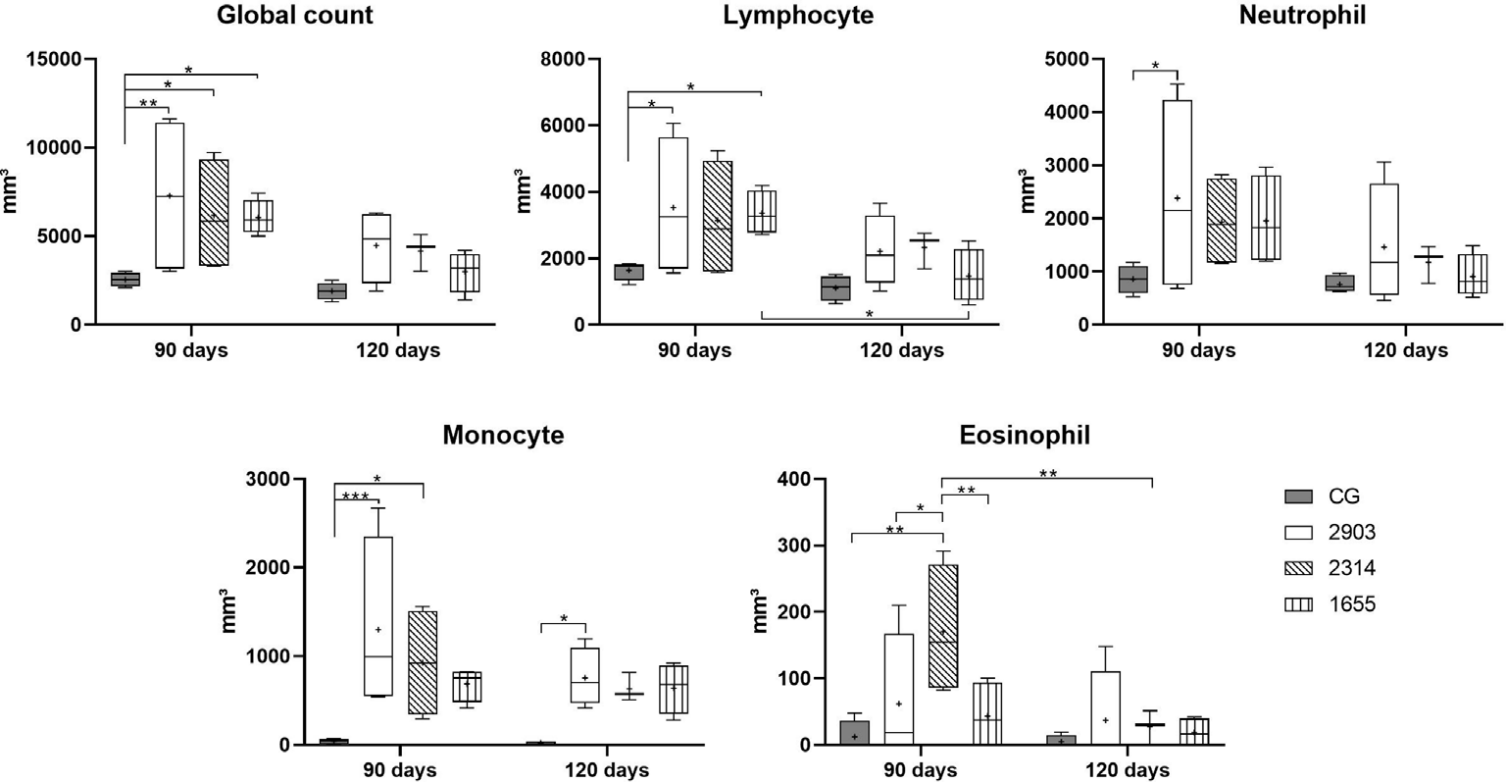
Global and differential leucocyte counts in peripheral blood in hamsters infected with three strains of *L. (V.) braziliensis* for 90 and 120 days. Data is represented in box plots (median with 25 to 75 percentile), whiskers (2.5 to 97.5 percentile), and mean (+) (*n* = 4). *p < 0.05; **p < 0.01; ***p < 0.001. CG, control group. 2903: group infected with MHOM/BR/1975/M2903. 2314: group infected with MHOM/BR/2003/2314. 1655: group infected with MHOM/BR/2000/1655.

Global leukocyte counts increased in all infected groups (90 days) when compared to control group (2903: *p* = 0.007; 2314: *p* = 0.033; 1655: *p* = 0.039). In differential leukocyte counts, we observed an increase in neutrophils (*p* = 0.029), lymphocytes (*p <* 0.03), and monocytes (*p <* 0.001) in the 2903 group at 90 days of infection when compared to the control group. When compared to the control group, monocyte quantities were also significantly higher at 120 days of infection in the 2903 group (*p* = 0.034) and 90 days in the 2314 group (*p* = 0.012); 1655 group presented *p* = 0.058 when compared to the CG. Lymphocytes also increased in the 1655 group at 90 days of infection when compared to the control group (*p* = 0.046); but lymphocyte numbers decreased between 90 and 120 days of infection for this group (1655; *p* = 0.029) (**Figure 3**).

### After Infection, Intestinal Walls Were Thicker, Crypts Were Deeper and Wider, and Villi Were Longer and Wider

**Figure 4:**
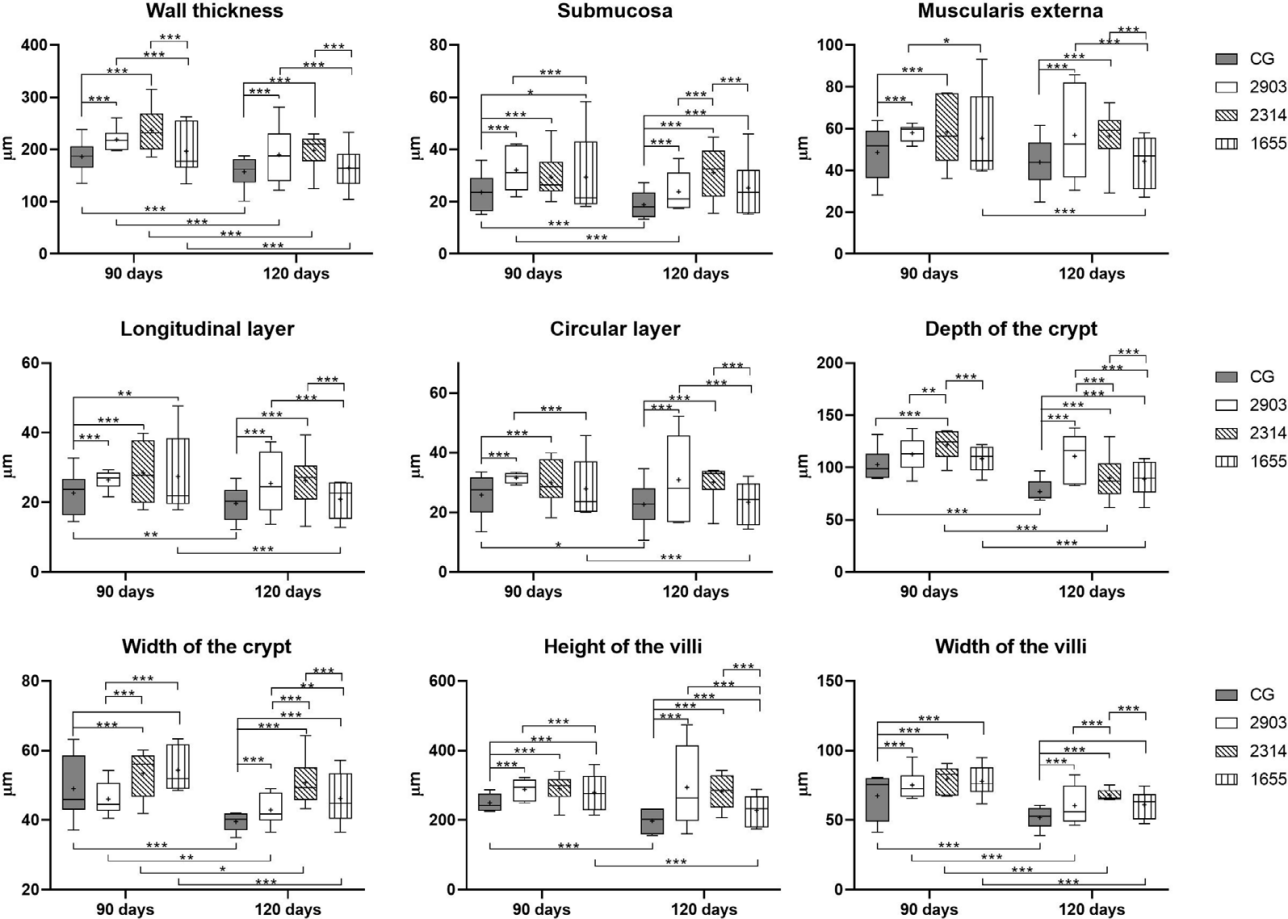
Morphometry of wall, villi, and crypts of ileums of hamsters infected by *L. (V.) braziliensis* for 90 or 120 days (µm). Data represented in box plots (median with 25 to 75 percentile), whiskers (2.5 to 97.5 percentile), and mean (+) (*n* = 6). *p < 0.05; **p < 0.01; ***p <  0.001. CG, control group. 2903: group infected with MHOM/BR/1975/M2903. 2314: group infected with MHOM/BR/2003/2314. 1655: group infected with MHOM/BR/2000/1655.

**Figure 5:**
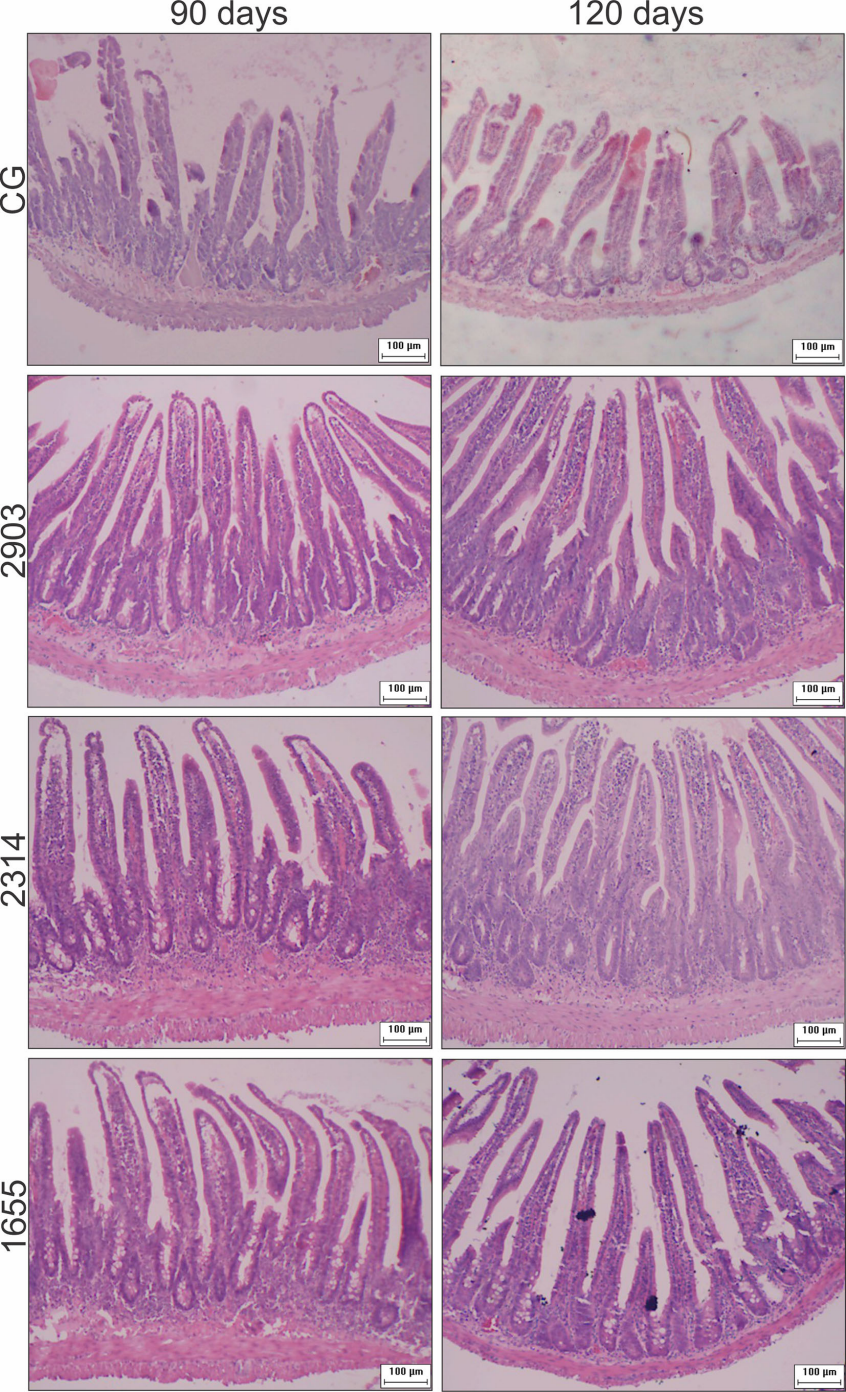
Photomicrograph of cross-sections representing the ileum wall, villi, and crypts of hamsters infected by *L. (V.) braziliensis* (HE staining, 10× magnification, scale bar = 100 µm, Olympus CX31). CG, control group. 2903: group infected with MHOM/BR/1975/M2903. 2314: group infected with MHOM/BR/2003/2314. 1655: group infected with MHOM/BR/2000/1655.

Ileums showed no significant macroscopic changes after infection. However, we measured intestinal walls to analyze the morphology of the organ and significant changes were found. We observed thicker muscular layers in groups 2903 and 2314 in both infection periods when compared to the control groups (*p <* 0.001). In groups 2903 and 2314, increases of approximately 20% and 29% of total muscle thickness were observed at 90 and 120 days of infection, respectively, when compared to the control groups. Group 1655 had values similar to the control group, with the exception of an increase to longitudinal layers at 90 days (*p* = 0.003). Submucosal layers were thicker in all infected groups and increased by an average of 25% at 90 days (2903 and 2314: *p <* 0.001; 1655: *p* = 0.023) and 42% at 120 days of infection (*p <* 0.001) and when compared to the control groups (**Figures 4** and **5**).

Crypts of group 2314 in the two infection periods and groups 2903 and 1655 at 120 days were deeper and wider than in control group hamsters (*p <* 0.001). Total organ wall size in groups 2903 and 2314 increased in both experimental periods when compared to the control groups (*p <* 0.001). We recorded an average increase of 16% and 35% in height and 15% and 22% in width at 90 and 120 days of infection, respectively, when compared to the control groups (*p <* 0.001). Thus, infection duration seemed to be a determining factor in organ response (**Figures 4** and **5**).

### The Infection Caused Morphometric and Quantitative Changes to Epithelial Cells in Hamster Ileums

**Figure 6:**
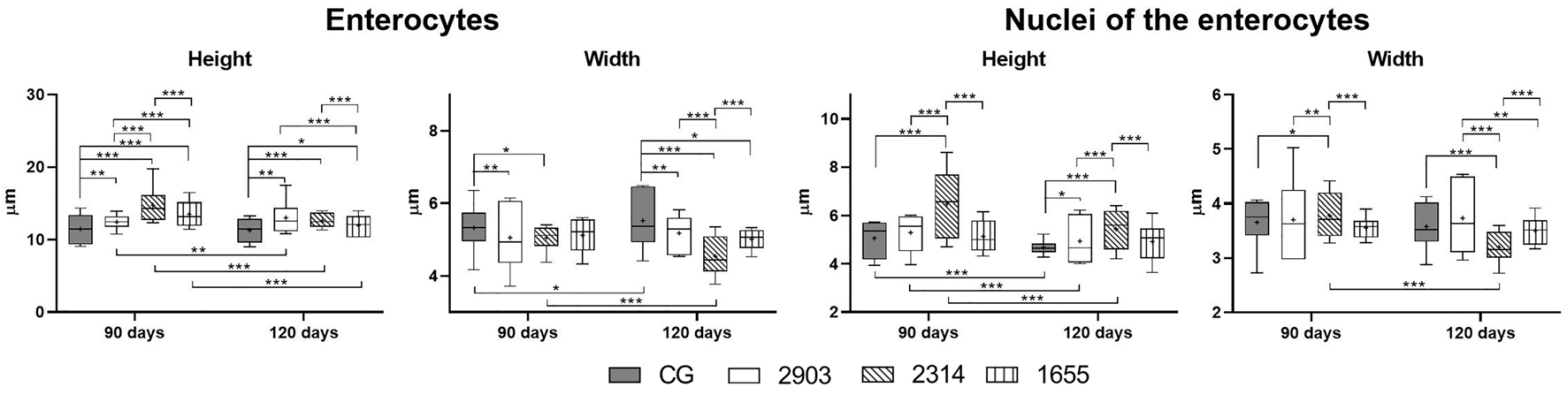
Morphometry of ileum enterocytes from hamsters infected with different *L.* (*V.) braziliensis* strains at 90 or 120 days of infection (µm). Data represented in box plots (median with 25 to 75 percentile), whiskers (2.5 to 97.5 percentile), and mean (+) (*n* = 6). *p <  0.05; **p <  0.01; ***p <  0.001. CG, control group. 2903: group infected with MHOM/BR/1975/M2903. 2314: group infected with MHOM/BR/2003/2314. 1655: group infected with MHOM/BR/2000/1655.

We measured enterocytes to verify the impacts of the infection on epithelial cells in the ileum. Enterocytes are the most abundant cell type in the intestinal epithelium. As shown in **Figure 6**, enterocytes increased in height (*p <* 0.05) and decreased in width (*p <* 0.05) in all infected groups when compared to the controls. We observed increases to the nuclei of these cells only in group 2314 (in both infection periods) when compared to the respective controls (*p <* 0.001). For group 2314, the longer infection period (120 days) resulted in a 29% increase to nuclei size when compared to the 90-day group (**Figure 6**).

**Figure 7:**
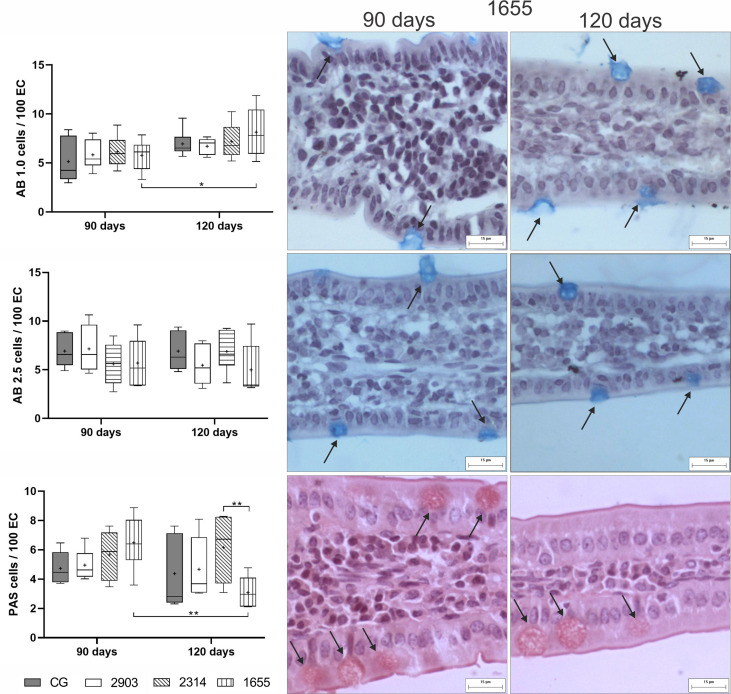
Ratio of globlet cells/100 of epithelial cells (EC) stained using the histochemical techniques of Alcian Blue (AB) pH 1.0, AB pH 2.5, and periodic acid-Schiff (PAS). Data is represented in box plots (median with 25 to 75 percentile), whiskers (2.5 to 97.5 percentile), and mean (+) (*n* = 6). *p <  0.05; **p <  0.01. Representative photomicrograph of the group 1655 at 90- and 120-days post infection showing goblet cells (black arrows) in each technique (AB pH 1.0 and 2.5 and PAS staining, 40× magnification, scale bar = 15 µm, Olympus BX50). CG, control group. 2903: group infected with MHOM/BR/1975/M2903. 2314: group infected with MHOM/BR/2003/2314. 1655: group infected with MHOM/BR/2000/1655.

In goblet cell quantification, we observed a 41% increase in the number of sulfomucin producers (AB 1.0; *p* = 0.028) and a 38% reduction in the neutral producers (PAS; *p* = 0.003) in group 1655 that were infected for 120 days when compared to the 90-day group. The 1655 group reduced the PAS goblet cells when compared to the 2314 group infected by 120 days (*p* = 0.007). No alterations were observed in the goblet cells that produce sialomucins (AB 2.5; *p >* 0.1) (**Figure 7**).

### Intraepithelial Lymphocytes and TGF-β-Immunoreactive Cells Increased After Infection

**Figure 8:**
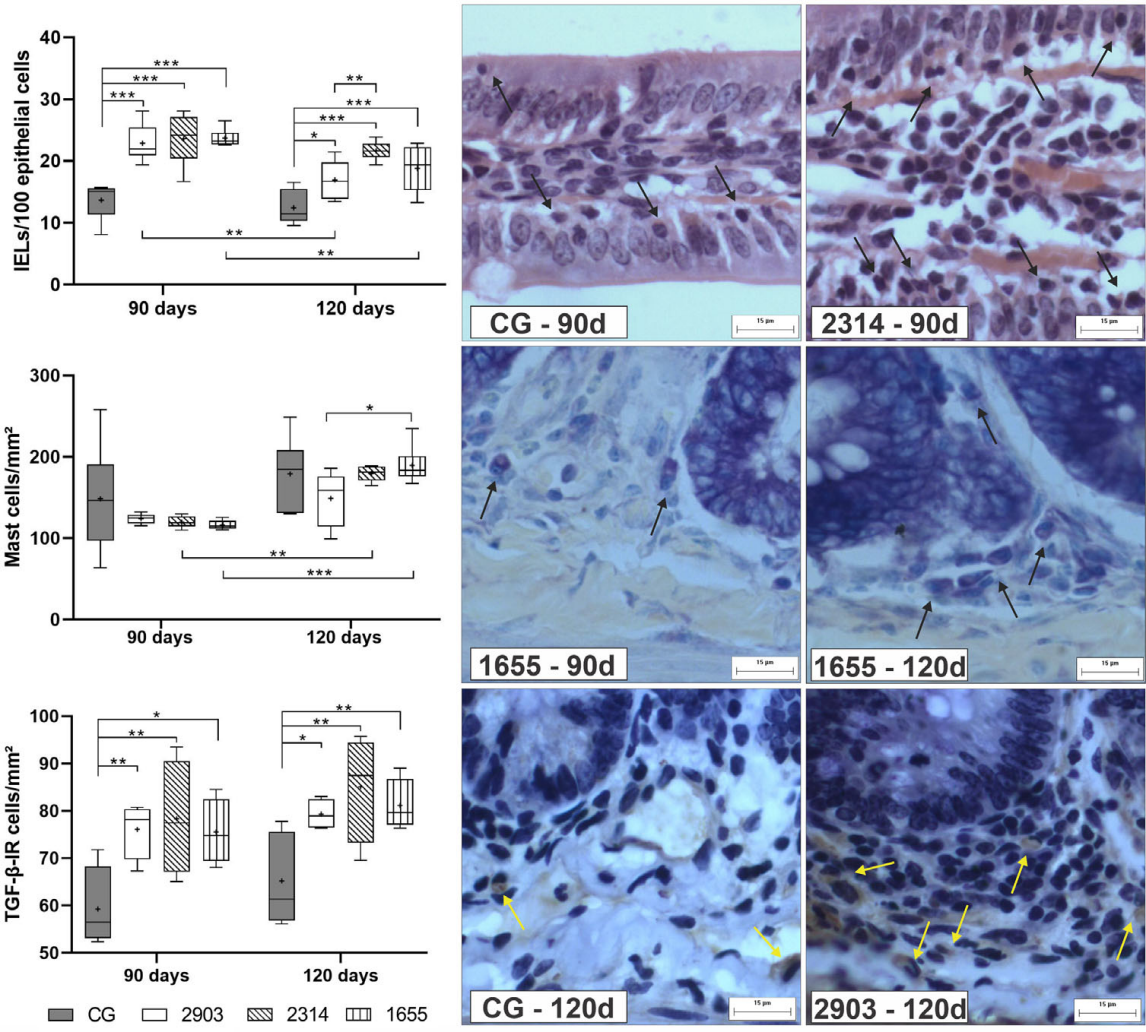
Proportion of intraepithelial lymphocytes (IELs) in 100 epithelial cells, mast cells, and TGF-β-immunoreactive cells (TGF-β-IR) per mm². Data represented in box plots (median with 25 to 75 percentile), whiskers (2.5 to 97.5 percentile), and mean (+) (*n* = 6). *p <  0.05; **p <  0.01; ***p <  0.001. Representative photomicrograph of IELs in the control group and 2314 at 90-days post infection (black arrows; HE staining, 40× magnification, scale bar = 15 µm, Olympus BX50); mast cells in group 1655 at 90- and 120-days post infection (black arrows; blue toluidine staining, 40× magnification, scale bar = 15 µm, Olympus BX50); TGF-β-IR of the control group and 2903 at 120-days post infection (yellow arrows; immunohistochemistry, 40× magnification, scale bar = 15 µm, Olympus BX50). CG, control group. 2903: group infected with MHOM/BR/1975/M2903. 2314: group infected with MHOM/BR/2003/2314. 1655: group infected with MHOM/BR/2000/1655.

When compared to the control, we observed an average increase of IELS of approximately 70% in the groups infected for 90 days (*p <* 0.001) and of 36%, 74%, and 51% in hamsters infected for 120 days in groups 2903 (*p* = 0.013), 2314 (*p <* 0.001), and 1655 (*p <* 0.001), respectively. Mast cell quantities significantly increased in the 2314 (*p* = 0.002) and 1655 (*p <* 0.001) groups at 120 days of infection when compared to 90 days. The number of TGF-β-IR cells increased in all infected groups when compared to the control groups (90 days – 2903: *p* = 0.009; 2314: *p* = 0.003; 1655: *p* = 0.012; 120 days – 2903: *p* = 0.020; 2314: *p* = 0.001; 1655: *p* = 0.009). The two-way ANOVA analyses revealed a significant main effect for the time [F_(1,40)_ = 16.5, *p <* 0.001] and infection [F_(3,40)_ = 24.2, *p <* 0.001] in IELs; for the time [F_(1,40)_ = 26.3, *p <* 0.001] in mast cells; and for the infection [F_(3,25)_ = 9.02, *p <* 0.001] in the TGF-β-IR cells (**Figure 8**).

### Infection and Experimental Duration Affect Collagen Fiber Remodeling

**Figure 9:**
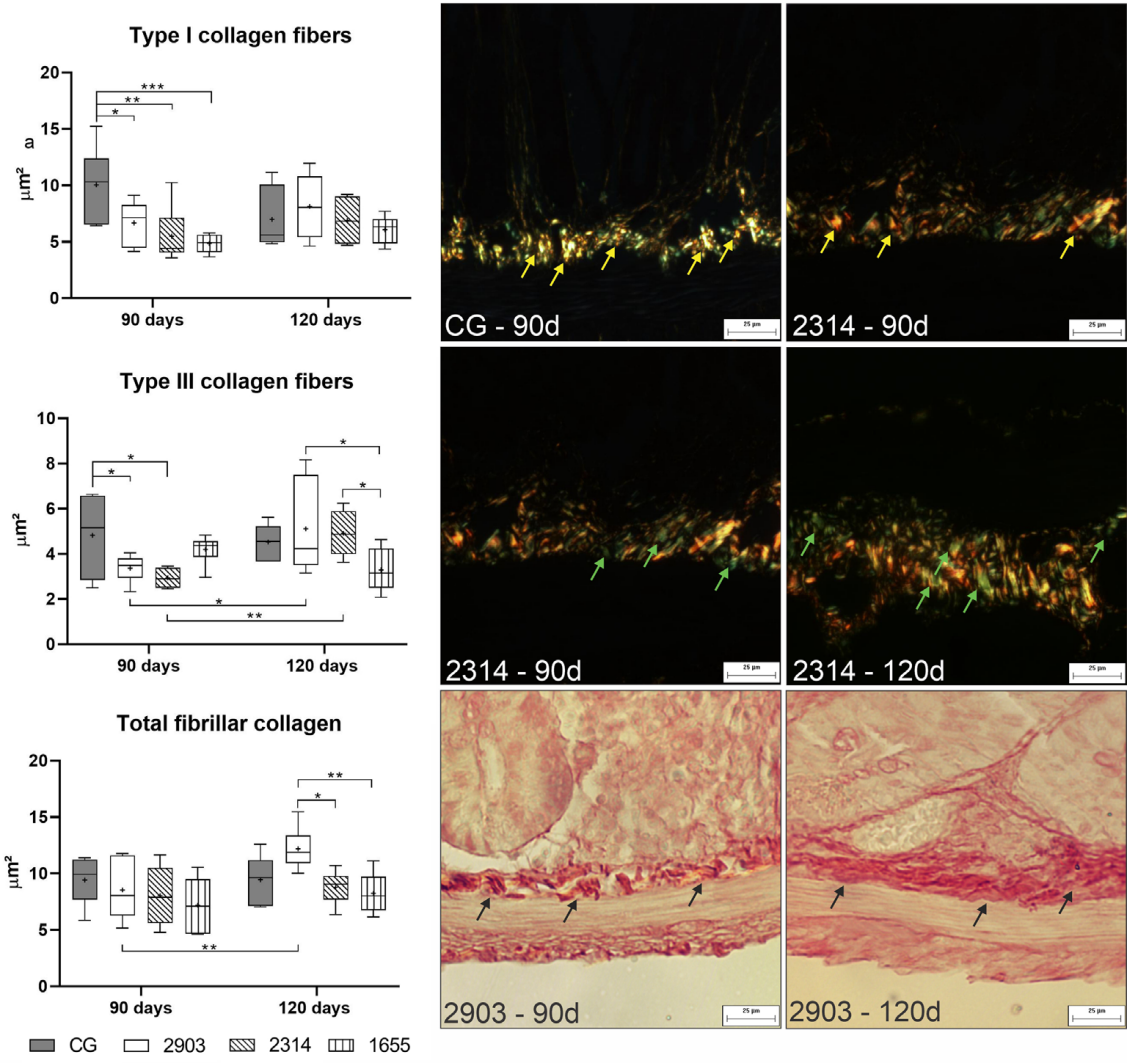
Area occupied by type I, type III, and total fibrillar collagen fibers (μm^2^). Data represented in box plots (median with 25 to 75 percentile), whiskers (2.5 to 97.5 percentile), and mean (+) (*n* = 6). *p <  0.05; **p <  0.01; ***p <  0.001. Photomicrograph of the area occupied by type I collagen fibers (yellow arrow) in control group and 2314 at 90-days post infection; by type III collagen fibers (green arrow) in group 2314 at 90- and 120-days post infection; and of total fibrillar collagen (black arrow) in group 2903 at 90- and 120-days post infection (Picrosirius Red staining, 20× magnification, scale bar = 25 µm, Olympus BX50). CG, control group. 2903: group infected with MHOM/BR/1975/M2903. 2314: group infected with MHOM/BR/2003/2314. 1655: group infected with MHOM/BR/2000/1655.

To understand the effects of the infection on the ileum cellular matrix, we used histochemistry to evaluate the areas occupied by collagen fibers. Hamsters from group 2903 showed higher total fibrillar collagen at 120 days of infection than at 90 days (*p <* 0.001). At 120 days of infection, group 2903 had higher total fibrillar collagen than all other groups in the experiment (*p <* 0.001). The two-way ANOVA demonstrated a significant main effect for the time [F_(1,40)_ = 4.70, *p* = 0.036] and infection [F_(3,40)_ = 3.35, *p* = 0.028] without interaction between the variables. At 90 days of infection, type I collagen fiber decreased in 2903 (*p* = 0.015), 2314 (*p* = 0.001) and 1655 (*p <* 0.001) groups when compared to the control. Hamsters in groups 2903 (*p* = 0.039) and 2314 (*p* = 0.012) had reductions in type III collagen fiber at 90 days of infection when compared to the controls, but type III fibers increased for both groups in the period between 90 to 120 days of infection (2903: *p* = 0.015; 2314: *p* = 0.008). In the type III collagen fibers was observed the interaction between the variables [F_(3,40)_ = 4.34, *p* = 0.009] by two-way ANOVA; while the type I demonstrated a significant main effect only for the infection [F_(3,40)_ = 4.06, *p* = 0.013] (**Figure 9**).

### Presence of Amastigotes and Inflammatory Changes in Ileums of Infected Hamsters

As demonstrated in **Table 1** after 120 days of infection, NO levels in hamster ileums in group 1655 were higher than in the control group. We observed an increase in myeloperoxidase enzyme activity in group 2903 when compared to group 1655 at 90 days of infection; the opposite was observed for NAG enzymatic activity.

**Table 1:** Biochemical analyses of the ileums of hamsters infected with different *L.* (*V.) braziliensis* strains at 90 and 120 days of infection.

		NO (μM)	MPO (OD)	NAG (OD/g of wet tissue)
	**CG**	47.41 ± 8.03	0.19 ± 0.02	23.23 ± 0.53
**90**	**2903**	57.25 ± 4.59	(0.24 ± 0.02)	(19.37 ± 2.01)
**days**	**2314**	68.29 ± 8.72	0.20 ± 0.03	25.17 ± 2.52
	**1655**	58.31 ± 13.27	(0.14 ± 0.02)^#^	(28.52 ± 1.85)^#^
	**CG**	(31.02 ± 6.20)	0.12 ± 0.02	17.87 ± 2.72
**120**	**2903**	51.32 ± 12.85	0.17 ± 0.03	21.71 ± 4.13
**days**	**2314**	38.76 ± 13.26	0.15 ± 0.06	22.28 ± 2.25
	**1655**	(66.51 ± 17.34)*	0.15 ± 0.03	21.76 ± 2.42

The data are expressed as mean ± SE (n = 4). *p < 0.05 comparing 1665 to CG at 120 days. ^#^p < 0.05 comparing 1655 to 2903 at 90 days. CG, control group. 2903: group infected with MHOM/BR/1975/M2903. 2314, group infected with MHOM/BR/2003/2314. 1655, group infected with MHOM/BR/2000/1655. NO, nitric oxide dosage; MPO, enzymatic activity of myeloperoxidase; NAG, enzymatic activity of N-acetyl-β-D-glucosaminidase; OD, optical density.

**Figure 10:**
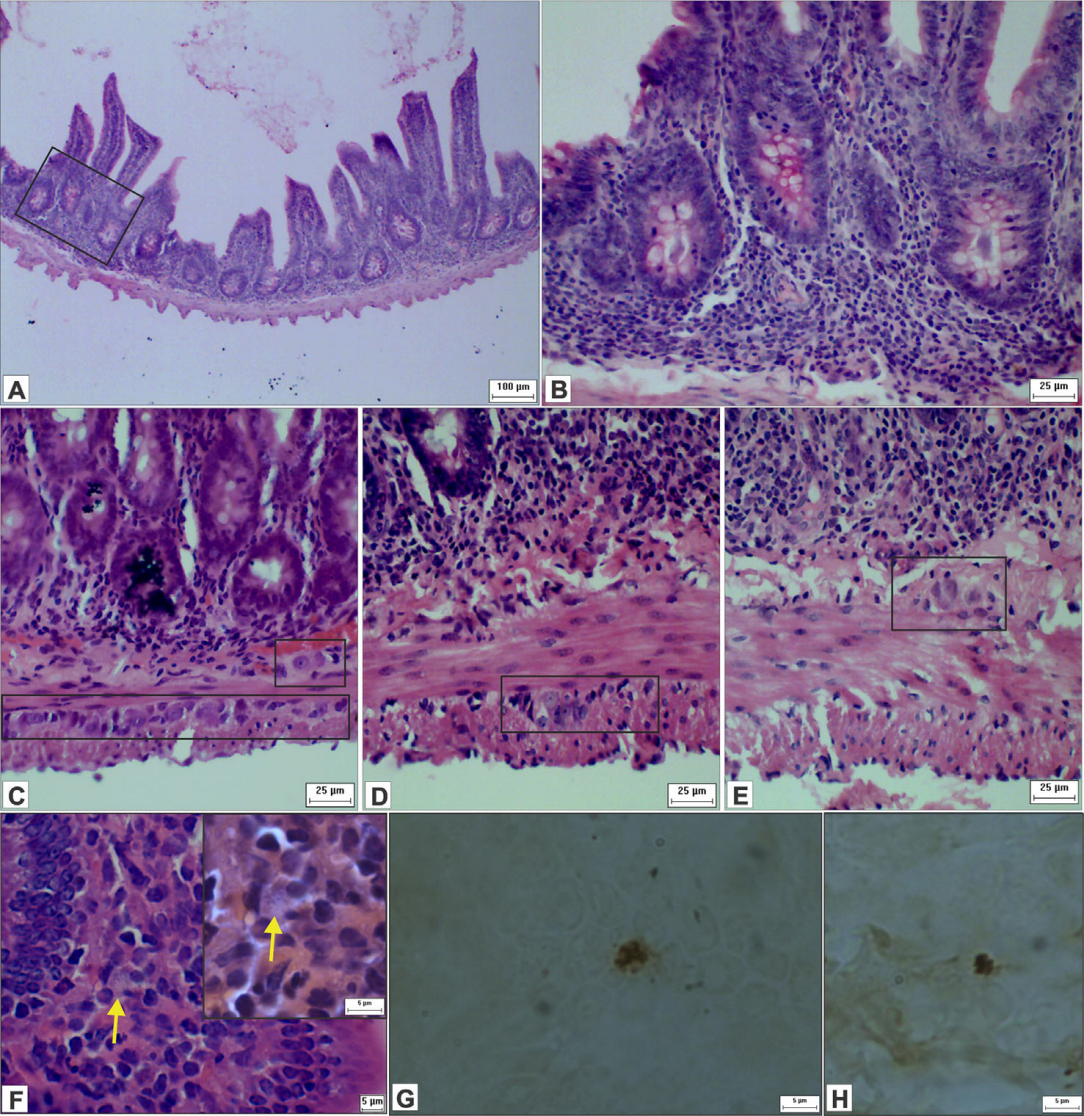
Photomicrograph of cross-sections of the ileum of hamsters infected by *L. (V.) braziliensis*. **(A)** Presence of inflammatory infiltrates in the intestinal wall and in the crypts (HE staining, 10× magnification, scale bar = 100 µm; Olympus CX31). **(B)** Higher magnification of image A (HE staining, 40× magnification, scale bar = 25 µm; Olympus CX31). **(C–E)** Inflammatory cells within and close to the myenteric and submucosal ganglia, suggestive of ganglionitis and periganglionitis (HE staining, 40× magnification, scale bar = 25 µm; Olympus CX31). **(F)** Suggestive amastigotes forms (arrow), presence of immune cells and IELs (HE staining, 100× magnification, scale bar = 5 µm; Olympus CX31) with a higher magnification (HE staining, 100× magnification, scale bar = 5 µm; Olympus BX50). **(G, H)** *Leishmania* amastigotes in the ileum (immunohistochemistry, 100× magnification, scale bar = 5 µm; Olympus BX50).

**Figure 10** shows an infiltration of mononuclear cells in the mucous layer formed mostly by lymphocytes, plasmocytes, and (to a lesser extent) polymorphonuclear leukocytes. In addition, we observed signs of inflammatory infiltrates in mucosa, submucosa and in crypts, and edemas in villi. Our findings of immune cells inside and around ganglia suggest that ganglionitis and periganglionitis could be correlated with changes observed in the ENS. While using hematoxylin and eosin staining, we also found forms that suggested amastigote presence; this presence was later confirmed by immunohistochemistry.

### The Infection Caused Morphometric Changes in Neurons in the Myenteric and Submucosal Plexuses

No quantitative changes in neurons or ganglias were observed in the evaluation of total neuronal populations (immunostained analysis with HuC/HuD) (**Table 2**).

**Table 2:** Counting of neurons in the myenteric and submucosal plexuses (marked by HuC/HuD) in the ileum of hamsters infected with different *L.* (*V.) braziliensis* strains at 90 and 120 days of infection.

		Myenteric neurons (mm²)	Submucosal plexus (mm²)			
			Number of ganglia	Neurons inside ganglia	Neurons outside ganglia	Submucous neurons
	**CG**	239.22 ± 4.62	9.13 ± 0.55	45.26 ± 3.27	15.43 ± 0.44	60.70 ± 3.61
**90**	**2903**	238.96 ± 5.54	11.10 ± 0.40	54.11 ± 1.04	15.26 ± 0.58	69.37 ± 0.87
**days**	**2314**	241.59 ± 5.00	10.12 ± 0.15	47.98 ± 1.72	14.80 ± 1.02	62.78 ± 1.45
	**1655**	229.74 ± 1.12	10.87 ± 0.38	51.45 ± 1.50	15.32 ± 0.72	66.77 ± 2.05
	**CG**	243.27 ± 3.08	9.94 ± 0.25	49.60 ± 0.79	16.36 ± 0.11	65.96 ± 0.87
**120**	**2903**	245.66 ± 11.40	9.40 ± 0.07	48.40 ± 0.90	16.49 ± 0.07	64.90 ± 0.88
**days**	**2314**	250.83 ± 7.28	10.02 ± 0.78	48.33 ± 3.26	14.88 ± 0.65	63.21 ± 3.91
	**1655**	242.65 ± 2.67	10.86 ± 0.35	50.72 ± 1.35	14.56 ± 0.58	65.28 ± 1.93

The data are expressed as mean ± SE in 1 mm^2^ (n = 4). GC, control group. 2903, group infected with MHOM/BR/1975/M2903. 2314, group infected with MHOM/BR/2003/2314. 1655, group infected with MHOM/BR/2000/1655. Number of neuron totals were counted in the myenteric plexus. Number of ganglia, the quantity of neurons inside and outside the ganglia, and the total number of neurons in the plexus were counted in the submucosal plexus.

**Figure 11:**
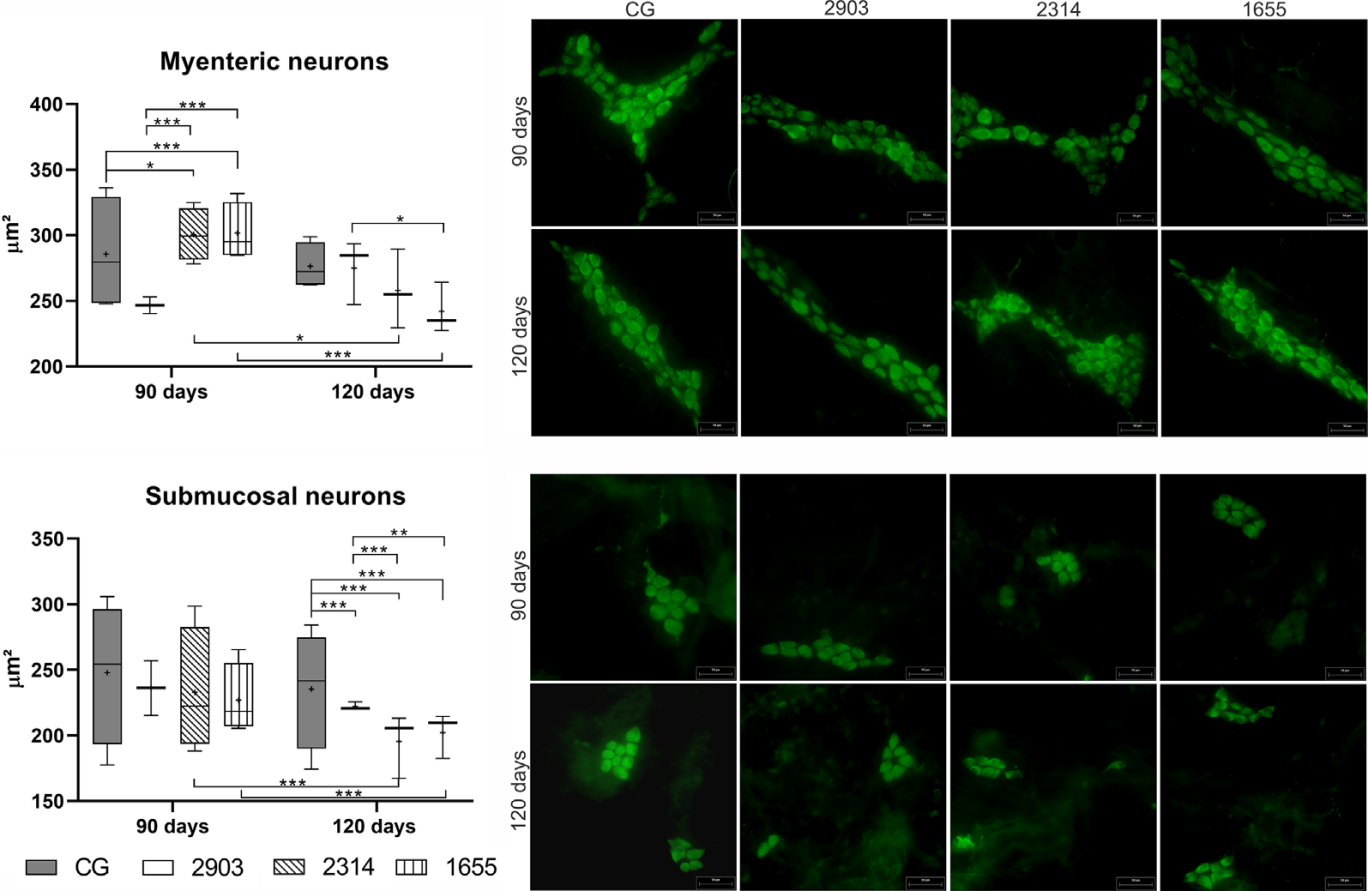
Size of neuron bodies (µm²) in the myenteric and submucosal plexuses of ileums of hamsters infected by *L. (V.) braziliensis* for 90 or 120 days. Data represented in box plots (median with 25 to 75 percentile), whiskers (2.5 to 97.5 percentile), and mean (+) (*n* = 4). *p <  0.05; **p <  0.01; ***p <  0.001. Photomicrograph of neurons from both plexuses (HuC/HuD immunohistochemistry, 20× magnification, scale bar = 50 µm, Olympus FSX100). CG, control group. 2903: group infected with MHOM/BR/1975/M2903. 2314: group infected with MHOM/BR/2003/2314. 1655: group infected with MHOM/BR/2000/1655.

In the myenteric plexus, the bodies of neurons in groups 2314 (*p* = 0.023) and 1655 (*p <* 0.001) at 90 days of infection were larger than the controls. However, significant reductions to cellular bodies were recorded thereafter (2314: *p* = 0.014; 1655: *p <* 0.001); at 120 days after infection, the cellular body sizes of infected groups were similar to the control groups. However, neuronal body sizes in the submucosal plexus at 120 days of infection decreased by approximately 15% in group 2903, 30% in 2314, and 26% in 1655 when compared to the 120 days control group (*p <* 0.001) (**Figure 11**).

## Discussion

Due to the clinical/epidemiological importance (Ministério da Saúde, 2017; Conceição-Silva and Morgado, 2019; World Health Organization, 2020) of the disease and its ability to use genetic diversity to create more aggressive forms (Guimarães et al., 2016; Rugani et al., 2018), our study evaluated the changes in hamster ileums at 90 days and 120 days of infection from one reference parasite strain and two clinically-isolated strains.

In infection-related lesions, different *L. (V.) braziliensis* strains have presented different biological behaviors (Rêgo et al., 2018) and cytokine and chemokine gene expressions in skin lesions in hamsters (Rêgo et al., 2019). For example, in BALB/c and C57BL/6 mice, different profiles of inflammatory infiltrates in livers and spleens were found with or without the presence of parasite (Pereira et al., 2009). Fernandes et al. (2016) demonstrated various cytokine expressions and macrophage-infectivity rates in *in vitro* experiments with the same strains used in our study.

Almeida and collaborators Almeida et al. (1996) reported the presence of *L. (V.) braziliensis* species in the spleens and livers of hamsters that could multiply *in situ*, which enables the production of secondary metastatic visceral lesions from a primary skin lesion (the reverse could also be possible). We found some *Leishmania* amastigote forms in infected hamster ileums with all strains analyzed in our study. The changes observed in this specific organ cannot be solely linked to the presence of the protozoan, but as a systemic consequence of infection. Pereira et al. (2009) also reported changes to livers and spleens of mice infected with different *L. (V.) braziliensis* strains, and Passos and collaborators Passos et al. (2020) demonstrated changes to hamster colons infected with *L. infantum*.

Increases of blood leukocytes confirmed the systemic effects of infection and necessity for mobilization of these cells to the site of initial infection and possibly other organs, such as the intestine. The presence of inflammatory infiltrates and increases to submucosa, muscular layers, and villi in the present study and other research (Góis et al., 2016; Santos et al., 2018a; Santos et al., 2018b) support this hypothesis. These effects enable the presence of the parasite in other organs, as defense cells maintain viable parasitic forms in the interior and are used as vehicles of the parasite to infect macrophages (Bogdan, 2020). When migrating from the infection site to the lymph nodes, macrophages and other immune cells can transport the parasite to other organs *via* the lymphatic system, as evidenced by the presence of amastigote forms in the popliteal lymph and the DNA of the parasite in mesenteric lymph nodes of hamsters chronically-infected with *L. (V.) braziliensis* (Santos et al., 2018b).

The inflammatory infiltrates observed in the lamina propria of infected hamsters mainly contained mononuclear cells, similar to observed in the duodenum of a patient infected by *L. donovani* (Chattopadhyay et al., 2020). At 120 days of infection, decreases were observed in various parameters of the intestinal wall and blood leukocytes when compared to groups infected for 90 days. This might be a natural part of the aging process, as this reduction was also seen in the control groups.

Ganglionitis and periganglionitis have been reported in hamsters infected with *L. (V.) braziliensis* (Santos et al., 2018b) and *L. infantum* (Passos et al., 2020), but our article is the first to numerically and morphometrically evaluate the neurons of both ENS plexuses. The infiltration of immune cells in the ganglia can lead to neuronal degeneration and consequently cause cell dysfunction and loss (De Giorgio et al., 2004). However, the neuroplastic response to inflammation enables intestinal homeostasis (Mawe et al., 2009).

Hypertrophy of neuron bodies in the myenteric plexus at 90 days of infection suggests an increase in their metabolic activity; this acts as a mechanism of adaptation to adverse conditions suffered by these cells (Araújo, 2015; Trevizan et al., 2019). Such alterations can interfere with intestinal motility and consequently lead to an increase in muscle layers, as this plexus is responsible for the coordinating movements of intestinal relaxation and contraction (Nezami and Srinivasan, 2010; Machado et al., 2021). This relationship has been observed with other protozoa (Araújo, 2015; Machado et al., 2021). At 120 days of infection, we observed reduction in neuronal bodies and muscle layers in group 1655; this may be related to more acidic mucus, and consequently, more fluid and easier secretion (Góis et al., 2016).

The increase in immune cells and the presence of edemas in the mucosal and submucosal layers contributed to the thickening of both layers in the ileal wall. The migration of these cells may have contributed to reductions to the body sizes of submucosal neurons in all infected groups at 120 days of infection. These data corroborate with those of other authors (da Silva et al., 2017; Schneider et al., 2018) who demonstrated that submucosal neurons are directly affected by intestinal inflammation. The submucosal plexus controls the function of epithelial cells *via* the lumen (Nezami and Srinivasan, 2010), chemical stimuli, and distension of intestinal mucosa (Christofi, 2008). The loss of normal functions of these neurons can cause dysfunction in intestinal permeability and secretion. We evaluated the total population of neurons (marked by HuC/HuD); other markers can also be used to detect possible changes in specific neuronal populations and ENS components.

The increase in cellularity in the lamina propria occurred together with large increases of IELs, demonstrating the attraction of immune cells to the epithelium (Santos et al., 2018a; Santos et al., 2018b). This effect corroborates with the previously discussed changes in submucosal neurons. IELs can play a protective role on enterocytes in the maintenance of epithelial integrity (Hu and Edelblum, 2017), an effect typically associated with inflammatory intestinal diseases (Mahadeva et al., 2002; Ahn et al., 2014; Sergi et al., 2017). Passos and collaborators (2020) found similar results in hamster colons after four months of *L. infantum* infection. In infected hamsters, we observed deeper crypts, longer and larger villis, and an increase of TGF-β-IR cells (a cytokine that controls growth and differentiation of enterocytes) (Sangild et al., 2009). These effects suggest there was a large proliferation of epithelial cells (Trevizan et al., 2016; Santos et al., 2018b); this may have led to the changes observed in the absorptive cells.

TGF-β may have contrasting roles in intestinal inflammation (Feagins, 2010). It possibly maintains intestinal homeostasis (Troncone et al., 2018). On the other hand, increased levels of this cytokine are found in areas of active intestinal inflammation (Feagins, 2010). It also has a role in the susceptibility of infection by *L. (V.) braziliensis* (Barral-Netto et al., 1992; Barral et al., 1995) and other species of *Leishmania* (Li et al., 1999; Gantt et al., 2003; Saha et al., 2007; Farage Frade et al., 2011). TGF-β performs the negative regulation of various macrophase-related microbicidal functions (Gantt et al., 2003; de Oliveira and Brodskyn, 2012). This includes decreasing NO production (Vodovotz et al., 1993; Bogdan, 2020), which is one of the principal defense mechanisms against infection (Bogdan, 2020). *In vitro* studies using the same strains as our study have not detected NO production by macrophages (Fernandes et al., 2016).

TGF-β acts on mast cells and performs opposite roles that may induce chemotaxis (Caslin et al., 2018). It is also capable of inhibiting the proliferation and development of mast cells and suppressing their function and survival (Ryan et al., 2007; Fernando et al., 2013; Caslin et al., 2018). Mast cells are important for tissue regeneration, as they enable the remodeling of collagen fibers (Hamilton et al., 2014). A positive correlation exists between the quantity of mast cells and immature collagen fibers (Ribeiro et al., 2018), which can be observed in group 2314 at 120 days of infection. Mature collagen fibers decreased after infection, demonstrating tissue remodeling (Pastre et al., 2019).

In the experimental conditions used in our study, *L. (V.) braziliensis* infection caused distinct alterations dependent by strain and infection duration in hamster ileum. In fact, in group 1655, some changes observed at 90 days of infection, approached values of the control group at 120 days.Ileums generally seemed to adapt to infection, considering the reduction of inflammatory cells and increase of elements involving tissue regeneration at 120 days of infection. The changes observed in hamsters infected with the MHOM/BR/2000/1655 strain were milder, although this strain was isolated from a patient with a reactivation of a lesion that had been previously considered cured. The groups infected with the strain isolated from a patient effectively treated with Glucantime^®^ (MHOM/BR/2003/2314) presented a greater effect in the ileum histoarchitecture that remained at 120 days of infection. These results show the significance of the host and their specific response to infection by different strains of the parasite.

Our results revealed that *L. (V.) braziliensis* infection leads to different morphological, cellular, biochemical and ENS neurons changes in hamster ileum. Although clinical signs were not observed during the experimental period, our results show that the intestine is a possible target for future studies of the *L. (V.) braziliensis* host relationship. Further studies are needed to clarify the impacts of these changes on the function of the organ and the mechanisms involved in the process.

## Data Availability Statement

The raw data supporting the conclusions of this article will be made available by the authors, without undue reservation.

## Ethics Statement

The animal study was reviewed and approved by Ethical Committee on Animal Use of the Universidade Estadual de Maringá under protocol number 7587260416.

## Author Contributions

AS, AF, TS, DS, and GN-M contributed to the conception, experimental design and implementation of the study. AS, MS, EC, and LL contributed to methodology and analyses. AS and GN-M was responsible for interpretation of data. AS and GN-M wrote the article. All authors contributed to the article and approved the submitted version.

## Funding

This study was financed in part by the Coordenação de Aperfeiçoamento de Pessoal de Nível Superior - Brasil (CAPES) - Finance Code 001 and by the Conselho Nacional de Desenvolvimento Científico e Tecnológico - Brasil (CNPq) - Grant Number: 4226522016-4.

## Conflict of Interest

The authors declare that the research was conducted in the absence of any commercial or financial relationships that could be construed as a potential conflict of interest.

## References

[B1] Abbas A. Lichtman A. Pillai S. (2017). Cellular and Molecular Immunology. 9th ed (Philadelphia: Elsevier).

[B2] Ahn J. Y. Lee K. H. Choi C. H. Kim J. W. Lee H. W. Kim J. W.. (2014). Colonic Mucosal Immune Activity in Irritable Bowel Syndrome: Comparison With Healthy Controls and Patients With Ulcerative Colitis. Dig. Dis. Sci. 59, 1001–1011. 10.1007/s10620-013-2930-4 24282051

[B3] Almeida M. C. Cuba-Cuba C. A. Moraes M. A. P. Miles M. A. (1996). Dissemination of *Leishmania (Viannia) braziliensis* . J. Comp. Pathol. 115, 311–316. 10.1016/S0021-9975(96)80088-0 8923241

[B4] Araújo E.J.d. (2015). *Toxoplasma Gondii* Causes Death and Plastic Alteration in the Jejunal Myenteric Plexus. World J. Gastroenterol. 21:4829. 10.3748/wjg.v21.i16.4829 25944996 PMC4408455

[B5] Baba C. S. Makharia G. K. Mathur P. Ray R. Gupta S. D. Samantaray J. C. (2006). Chronic Diarrhea and Malabsorption Caused by *Leishmania Donovani* . Indian J. Gastroenterol. 25, 309–310. 17264434

[B6] Barral A. de Freitas L. A. R. Carvalho E. M. Almeida R. P. Barral-Netto M. de Jesus A. M. R. (1996). Biological Behavior of *Leishmania Amazonensis* Isolated From Humans With Cutaneous, Mucosal, or Visceral Leishmaniasis in Balb/C Mice. Am. J. Trop. Med. Hyg. 54, 178–184. 10.4269/ajtmh.1996.54.178 8619444

[B7] Barral-Netto M. Barral A. Brownell C. E. Skeiky Y. A. W. Ellingsworth L. R. Twardzik D. R.. (1992). Transforming Growth Factor-β in Leishmanial Infection: A Parasite Escape Mechanism. Sci. (80-. ) 257, 545–548. 10.1126/science.1636092 1636092

[B8] Barral A. Teixeira M. Reis P. Vinhas V. Costa J. Lessa H.. (1995). Transforming Growth Factor-Beta in Human Cutaneous Leishmaniasis. Am. J. Pathol. 147, 947–954. 7573370 PMC1871026

[B9] Bogdan C. (2020). Macrophages as Host, Effector and Immunoregulatory Cells in Leishmaniasis: Impact of Tissue Micro-Environment and Metabolism. Cytokine X 2:100041. 10.1016/j.cytox.2020.100041 33604563 PMC7885870

[B10] Caslin H. L. Kiwanuka K. N. Haque T. T. Taruselli M. T. MacKnight H. P. Paranjape A.. (2018). Controlling Mast Cell Activation and Homeostasis: Work Influenced by Bill Paul That Continues Today. Front. Immunol. 9, 868. 10.3389/fimmu.2018.00868 29755466 PMC5932183

[B11] Chassaing B. Kumar M. Baker M. T. Singh V. Vijay-Kumar M. (2014). Mammalian Gut Immunity. Biomed. J. 37, 246–258. 10.4103/2319-4170.130922 25163502 PMC4714863

[B12] Chattopadhyay A. Mittal S. Gupta K. Dhir V. Jain S. (2020). Intestinal Leishmaniasis. Clin. Microbiol. Infect. 26, 1345–1346. 10.1016/j.cmi.2020.05.003 32439594

[B13] Christofi F. L. (2008). Purinergic Receptors and Gastrointestinal Secretomotor Function. Purinergic Signal. 4, 213–236. 10.1007/s11302-008-9104-4 18604596 PMC2486343

[B14] Conceição-Silva F. Morgado F. N. (2019). *Leishmania* Spp-Host Interaction: There Is Always an Onset, But Is There An End? Front. Cell. Infect. Microbiol. 9, 330. 10.3389/fcimb.2019.00330 31608245 PMC6761226

[B15] Cruz A. A. V. Alves-Ferreira E. V. C. Milbratz-Moré G. Chahud F. Ruy P. C. Duarte M. I. S.. (2017). Case Report: Sclerosing Orbital Inflammation Caused by *Leishmania braziliensis* . Am. J. Trop. Med. Hyg. 96, 197–199. 10.4269/ajtmh.16-0389 27799649 PMC5239692

[B16] da Silva M. V. Marosti A. R. Mendes C. E. Palombit K. Castelucci P. (2017). Submucosal Neurons and Enteric Glial Cells Expressing the P2X7 Receptor in Rat Experimental Colitis. Acta Histochem. 119, 481–494. 10.1016/j.acthis.2017.05.001 28501138

[B17] De Giorgio R. Guerrini S. Barbara G. Stanghellini V. De Ponti F. Corinaldesi R.. (2004). Inflammatory Neuropathies of the Enteric Nervous System. Gastroenterology 126, 1872–1883. 10.1053/j.gastro.2004.02.024 15188182

[B18] de Oliveira C. I. Brodskyn C. I. (2012). The Immunobiology of *Leishmania braziliensis* Infection. Front. Immunol. 3:145. 10.3389/fimmu.2012.00145 22701117 PMC3370302

[B19] De Oliveira C. I. Teixeira M. J. Gomes R. Barral A. Brodskyn C. (2004). Animal Models for Infectious Diseases Caused by Parasites: Leishmaniasis. Drug Discovery Today Dis. Model. 1, 81–86. 10.1016/j.ddmod.2004.07.005

[B20] Drokhlyansky E. Smillie C. S. Van Wittenberghe N. Ericsson M. Griffin G. K. Eraslan G.. (2020). The Human and Mouse Enteric Nervous System at Single-Cell Resolution. Cell 182, 1606–1622.e23. 10.1016/j.cell.2020.08.003 32888429 PMC8358727

[B21] Farage Frade A. Campos de Oliveira L. Lamounier Costa D. Henrique Nery Costa C. Aquino D. Van Weyenbergh J.. (2011). TGFB1 and IL8 Gene Polymorphisms and Susceptibility to Visceral Leishmaniasis. Infect. Genet. Evol. 11, 912–916. 10.1016/j.meegid.2011.02.014 21376140

[B22] Feagins L. A. (2010). Role of Transforming Growth Factor-β in Inflammatory Bowel Disease and Colitis-Associated Colon Cancer. Inflamm. Bowel Dis. 16, 1963–1968. 10.1002/ibd.21281 20848467

[B23] Fernandes A. C. B. S. Pedroso R. B. de Mello T. F. P. Donatti L. Venazzi E. A. S. Demarchi I. G.. (2016). *In Vitro* Characterization of *Leishmania (Viannia) braziliensis* Isolates From Patients With Different Responses to Glucantime ® Treatment From Northwest Paraná, Brazil. Exp. Parasitol. 167, 83–93. 10.1016/j.exppara.2016.05.003 27181585

[B24] Fernando J. Faber T. W. Pullen N. A. Falanga Y. T. Kolawole E. M. Oskeritzian C. A.. (2013). Genotype-Dependent Effects of TGF-β1 on Mast Cell Function: Targeting the Stat5 Pathway. J. Immunol. 191, 4505–4513. 10.4049/jimmunol.1202723 24068671 PMC3846427

[B25] Figueiredo M. M. Deoti B. Amorim I. F. Pinto A. J. W. Moraes A. Carvalho C. S.. (2014). Expression of Regulatory T Cells in Jejunum, Colon, and Cervical and Mesenteric Lymph Nodes of Dogs Naturally Infected With *Leishmania Infantum* . Infect. Immun. 82, 3704–3712. 10.1128/IAI.01862-14 24935975 PMC4187817

[B26] Gagini T. De Oliveira Schubach A. De Fatima Madeira M. Valete-Rosalino C. M. Pimentel M. I. F. Da Silva Pacheco R. (2017). Genotypic Profiles of Leishmania (Viannia) braziliensis Strains From Cutaneous Leishmaniasis Patients and Their Relationship With the Response to Meglumine Antimoniate Treatment: A Pilot Study. Parasite 24, 1–11. 10.1051/parasite/2017035 28959938 PMC5621350

[B27] Gantt K. R. Schultz-Cherry S. Rodriguez N. Jeronimo S. M. B. Nascimento E. T. Goldman T. L.. (2003). Activation of TGF-β by *Leishmania Chagasi*: Importance for Parasite Survival in Macrophages. J. Immunol. 170, 2613–2620. 10.4049/jimmunol.170.5.2613 12594289

[B28] Góis M. B. Hermes-Uliana C. Barreto Zago M. C. Zanoni J. N. da Silva A. V. de Miranda-Neto M. H.. (2016). Chronic Infection With *Toxoplasma Gondii* Induces Death of Submucosal Enteric Neurons and Damage in the Colonic Mucosa of Rats. Exp. Parasitol. 164, 56–63. 10.1016/j.exppara.2016.02.009 26902605

[B29] Gomes-Silva A. Valverde J. G. Ribeiro-Romão R. P. Plácido-Pereira R. M. da-Cruz A. M. (2013). Golden Hamster (*Mesocricetus Auratus*) as an Experimental Model for *Leishmania (Viannia) braziliensis* Infection. Parasitology 140, 771–779. 10.1017/S0031182012002156 23369503

[B30] Gontijo C. M. Pacheco R. S. Oréfice F. Lasmar E. Silva E. S. Melo M. N. (2002). Concurrent Cutaneous, Visceral and Ocular Leishmaniasis Caused by *Leishmania (Viannia) braziliensis* in a Kidney Transplant Patient. Mem. Inst. Oswaldo Cruz 97, 751–753. 10.1590/S0074-02762002000500029 12219147

[B31] Guimarães L. H. Queiroz A. Silva J. A. Silva S. C. Magalhães V. Lago E. L.. (2016). Atypical Manifestations of Cutaneous Leishmaniasis in a Region Endemic for *Leishmania braziliensis*: Clinical, Immunological and Parasitological Aspects. PloS Negl. Trop. Dis. 10, e0005100. 10.1371/journal.pntd.0005100 27906988 PMC5131895

[B32] Hamilton M. J. Frei S. M. Stevens R. L. (2014). The Multifaceted Mast Cell in Inflammatory Bowel Disease. Inflamm. Bowel Dis. 20, 2364–2378. 10.1097/MIB.0000000000000142 25401721 PMC4428674

[B33] Hoyos C. L. Quipildor M. Bracamonte E. Lauthier J. J. Cajal P. Uncos A.. (2019). Simultaneous Occurrence of Cutaneous and Mucocutaneous Leishmaniasis Caused by Different Genotypes of *Leishmania (Viannia) braziliensis* . J. Dermatol. 46, e320–e322. 10.1111/1346-8138.14866 30938461

[B34] Hu M. D. Edelblum,. K. L. (2017). Sentinels at the Frontline: The Role of Intraepithelial Lymphocytes in Inflammatory Bowel Disease. Curr. Pharmacol. Rep. 3, 321–334. 10.1007/s40495-017-0105-2 29242771 PMC5724577

[B35] Jacobson A. Yang D. Vella M. Chiu,. I. M. (2021). The Intestinal Neuro-Immune Axis: Crosstalk Between Neurons, Immune Cells, and Microbes. Mucosal Immunol. 14, 555–565. 10.1038/s41385-020-00368-1 33542493 PMC8075967

[B36] Lewis M. D. Paun A. Romano A. Langston H. Langner C. A. Moore I. N.. (2020). Fatal Progression of Experimental Visceral Leishmaniasis is Associated With Intestinal Parasitism and Secondary Infection by Commensal Bacteria, and is Delayed by Antibiotic Prophylaxis. PloS Pathog. 16, e1008456. 10.1371/journal.ppat.1008456 32282850 PMC7179947

[B37] Li J. Hunter C. A. Farrell J. P. (1999). Anti-TGF-Beta Treatment Promotes Rapid Healing of *Leishmania Major* Infection in Mice by Enhancing *In Vivo* Nitric Oxide Production. J. Immunol. 162, 974–979. 9916722

[B38] Machado C. C. A. Watanabe P. Mendes d. de L J. D. Pupim A. C. E. Ortigoza S. M.. (2021). *Toxoplasma Gondii* Infection Impairs the Colonic Motility of Rats Due to Loss of Myenteric Neurons. Neurogastroenterol. Motil. 33:13967. 10.1111/nmo.13967 32812313

[B39] Mahadeva S. Wyatt J. I. Howdle P. D. (2002). Is a Raised Intraepithelial Lymphocyte Count With Normal Duodenal Villous Architecture Clinically Relevant? J. Clin. Pathol. 55, 424–428. 10.1136/jcp.55.6.424 12037023 PMC1769667

[B40] Mawe G. M. Strong D. S. Sharkey K. A. (2009). Plasticity of Enteric Nerve Functions in the Inflamed and Postinflamed Gut. Neurogastroenterol. Motil. 21, 481–491. 10.1111/j.1365-2982.2009.01291.x 19368664 PMC2717558

[B41] Ministério da Saúde (2017). “Manual de Vigilância da Leishmaniose Tegumentar Americana,” 1st ed (Brasília: Ministério da Saúde). Available at: http://bvsms.saude.gov.br/bvs/publicacoes/manual_vigilancia_leishmaniose_tegumentar_americana.pdf.

[B42] Mowat A. M. Agace W. W. (2014). Regional Specialization Within the Intestinal Immune System. Nat. Rev. Immunol. 14, 667–685. 10.1038/nri3738 25234148

[B43] Nezami B. G. Srinivasan S. (2010). Enteric Nervous System in the Small Intestine: Pathophysiology and Clinical Implications. Curr. Gastroenterol. Rep. 12, 358–365. 10.1007/s11894-010-0129-9 20725870 PMC3752592

[B44] Pan American Health Organization (2019). Leishmaniasis: Epidemiological Report in the Americas (Washington, D.C). Available at: http://iris.paho.org/xmlui/handle/123456789/50505.

[B45] Panza S. B. Vargas R. Balbo S. L. Bonfleur M. L. Granzotto D. C. T. Sant’Ana D. M. G.. (2021). Perinatal Exposure to Low Doses of Glyphosate-Based Herbicide Combined With a High-Fat Diet in Adulthood Causes Changes in the Jejunums of Mice. Life Sci. 275, 119350. 10.1016/j.lfs.2021.119350 33737081

[B46] Passos F. C. Gois M. B. Sousa A. D. de Marinho A. I. L. Corvo L. Soto M.. (2020). Investigating Associations Between Intestinal Alterations and Parasite Load According to Bifidobacterium Spp. And Lactobacillus Spp. Abundance in the Gut Microbiota of Hamsters Infected by *Leishmania Infantum* . Mem. Inst. Oswaldo Cruz 115, 1–11. 10.1590/0074-02760200377 PMC7703327 33263602

[B47] Pastre M. J. Casagrande L. Gois M. B. Pereira-Severi L. S. Miqueloto C. A. Garcia J. L.. (2019). *Toxoplasma Gondii* Causes Increased ICAM-1 and Serotonin Expression in the Jejunum of Rats 12 H After Infection. Biomed. Pharmacother. 114:108797. 10.1016/J.BIOPHA.2019.108797 30951950

[B48] Patino L. H. Muñoz M. Cruz-Saavedra L. Muskus C. Ramírez J. D. (2020). Genomic Diversification, Structural Plasticity, and Hybridization in *Leishmania (Viannia) braziliensis* . Front. Cell. Infect. Microbiol. 10, 582192. 10.3389/fcimb.2020.582192 33178631 PMC7596589

[B49] Pereira C. G. Silva A. L. N. de Castilhos P. Mastrantonio E. C. Souza R. A. Romão R. P.. (2009). Different Isolates From *Leishmania braziliensis* Complex Induce Distinct Histopathological Features in a Murine Model of Infection. Vet. Parasitol. 165, 231–240. 10.1016/j.vetpar.2009.07.019 19656631

[B50] Quaresma P. F. De Brito C. F. A. Rugani J. M. N. Freire J. D. M. Baptista R. D. P. Moreno E. C.. (2018). Distinct Genetic Profiles of *Leishmania (Viannia) braziliensis* Associate With Clinical Variations in Cutaneous-Leishmaniasis Patients From an Endemic Area in Brazil. Proc. Int. Astron. Union 145, 1161–1169. 10.1017/S0031182018000276 29526166

[B51] Raina S. Raina R. K. Bodh A. Rana B. S. Sharma R. (2017). Gastrointestinal Leishmaniasis in Non-Endemic Region. J. Assoc. Phys. India 65, 106–107. 28782325

[B52] Rêgo F. D. da Rocha Lima A. C. V. M. Pereira A. A. S. Quaresma P. F. Pascoal-Xavier M. A. Shaw J. J.. (2018). Genetic Variant Strains of *Leishmania (Viannia) braziliensis* Exhibit Distinct Biological Behaviors. Parasitol. Res. 117, 3157–3168. 10.1007/s00436-018-6014-4 30022292

[B53] Rêgo F. D. Fradico J. R. B. Teixeira-Carvalho A. Gontijo C. M. F. (2019). Molecular Variants of *Leishmania (Viannia) braziliensis* Trigger Distinct Patterns of Cytokines and Chemokines Expression in Golden Hamster. Mol. Immunol. 106, 36–45. 10.1016/j.molimm.2018.12.013 30576950

[B54] Ribeiro L. S. F. dos Santos J. N. Rocha C. A. G. Cury P. R. (2018). Association Between Mast Cells and Collagen Maturation in Chronic Periodontitis in Humans. J. Histochem. Cytochem. 66, 467–475. 10.1369/0022155418765131 29553869 PMC5977442

[B55] Ribeiro-Romão R. P. Moreira O. C. Osorio E. Y. Cysne-Finkelstein L. Gomes-Silva A. Valverde J. G.. (2014). Comparative Evaluation of Lesion Development, Tissue Damage, and Cytokine Expression in Golden Hamsters (*Mesocricetus Auratus*) Infected by Inocula With Different *Leishmania (Viannia) braziliensis* Concentrations. Infect. Immun. 82, 5203–5213. 10.1128/IAI.02083-14 25287925 PMC4249292

[B56] Rugani J. N. Quaresma P. F. Gontijo C. F. Soares R. P. Monte-Neto R. L. (2018). Intraspecies Susceptibility of *Leishmania (Viannia) braziliensis* to Antileishmanial Drugs: Antimony Resistance in Human Isolates From Atypical Lesions. Biomed. Pharmacother. 108, 1170–1180. 10.1016/j.biopha.2018.09.149 30372818

[B57] Ryan J. J. Kashyap M. Bailey D. Kennedy S. Speiran K. Brenzovich J.. (2007). Mast Cell Homeostasis: A Fundamental Aspect of Allergic Disease. Crit. Rev. Immunol. 27, 15–32. 10.1615/critrevimmunol.v27.i1.20 17430094

[B58] Saha S. Mondal S. Ravindran R. Bhowmick S. Modak D. Mallick S.. (2007). IL-10- and TGF-β-Mediated Susceptibility in Kala-Azar and Post-Kala-Azar Dermal Leishmaniasis: The Significance of Amphotericin B in the Control of *Leishmania Donovani* Infection in India. J. Immunol. 179, 5592–5603. 10.4049/jimmunol.179.8.5592 17911647

[B59] Sangild P. T. Mei J. Fowden A. L. Xu R. J. (2009). The Prenatal Porcine Intestine has Low Transforming Growth Factor-Beta Ligand and Receptor Density and Shows Reduced Trophic Response to Enteral Diets. Am. J. Physiol. Integr. Comp. Physiol. 296, R1053–R1062. 10.1152/ajpregu.90790.2008 19158412

[B60] Santaolalla R. Fukata M. Abreu M. T. (2011). Innate Immunity in the Small Intestine. Curr. Opin. Gastroenterol. 27, 125–131. 10.1097/MOG.0b013e3283438dea 21248635 PMC3502877

[B61] Santos A.G.A.d. Ferlini J. de P. Vicentino S. L. Lonardoni M. V. C. Sant’Ana D. de M. G.. (2018a). Alterations Induced in the Ileum of Mice Upon Inoculation With Different Species of *Leishmania*: A Preliminary Study. Rev. Soc Bras. Med. Trop. 51, 537–541. 10.1590/0037-8682-0348-2017 30133641

[B62] Santos A.G.A.d. Lima L.L. de Mota C. A. Gois M. B. Fernandes A. C. B. S. Silveira T. G. V.. (2018b). Insights of *Leishmania (Viannia) braziliensis* Infection in Golden Hamster (*Mesocricetus Auratus*) Intestine. Biomed. Pharmacother. 106, 1624–1632. 10.1016/j.biopha.2018.07.120 30119238

[B63] Schneider L. C. L. do Nascimento J. C. P. Trevizan A. R. Góis M. B. Borges S. C. Beraldi E. J.. (2018). *Toxoplasma Gondii* Promotes Changes in VIPergic Submucosal Neurons, Mucosal Intraepithelial Lymphocytes, and Goblet Cells During Acute Infection in the Ileum of Rats. Neurogastroenterol. Motil. 30, e13264. 10.1111/nmo.13264 29266818

[B64] Sergi C. Shen F. Bouma G. (2017). Intraepithelial Lymphocytes, Scores, Mimickers and Challenges in Diagnosing Gluten-Sensitive Enteropathy (Celiac Disease). World J. Gastroenterol. 23, 573–589. 10.3748/wjg.v23.i4.573 28216964 PMC5292331

[B65] Silva D.T. da Alves M. L. Spada J. C. P. Silveira R. de C.V. Oliveira T.M.F.d. Starke-Buzetti,. W. A. (2018). Neutrophils, Eosinophils, and Mast Cells in the Intestinal Wall of Dogs Naturally Infected With *Leishmania Infantum* . Rev. Bras. Parasitol. Vet. 27, 430–438. 10.1590/s1984-296120180085 30517421

[B66] Silva L. Damrose E. Fernandes, A.-M.-F. (2017). Laryngeal Leishmaniasis, a Rare Manifestation of an Emerging Disease. Eur. Ann. Otorhinolaryngol. Head Neck Dis. 134, 211–212. 10.1016/j.anorl.2015.11.013 28344078

[B67] Silva D. T. Neves M. F. Queiroz N. M. G. P. de Spada J. C. P. Alves M. L. Flóro e Silva M.. (2016). Correlation Study and Histopathological Description of Intestinal Alterations in Dogs Infected With Leishmania Infantum. Rev. Bras. Parasitol. Vet. 25, 24–36. 10.1590/S1984-29612016009 26982556

[B68] Silva E.S. da Pacheco R. S. Gontijo C. M. F. Carvalho I. R. Brazil R. P. (2002). Visceral Leishmaniasis Caused by *Leishmania (Viannia) braziliensis* in a Patient Infected With Human Immunodeficiency Virus. Rev. do Inst. Med. Trop. São Paulo 44, 145–149. 10.1590/S0036-46652002000300006 12163907

[B69] Soria López E. Olalla Sierra J. Del Arco Jiménez A. Pereda Salguero T. Abitei C. de la Torre Lima J. (2016). Colonic Leishmaniasis in a Patient With HIV: A Case Report. Rev. Esp. Enferm. Dig. 108, 838–840. 10.17235/reed.2016.4038/2015 26901148

[B70] Souza K. D. Fernandes E. P. A. Santos A. G. A. Lima L. L. Gonzaga W. F. K. M. Xander P.. (2019). Infection by *Leishmania (Leishmania) Infantum Chagasi* Causes Intestinal Changes B-1 Cells Dependent. Parasit. Immunol. 41, e12661. 10.1111/pim.12661 31267529

[B71] Trevizan A. R. Schneider L. C. L. Araújo E. J. de A. Garcia J. L. Buttow N. C.. (2019). Acute *Toxoplasma Gondii* Infection Alters the Number of Neurons and the Proportion of Enteric Glial Cells in the Duodenum in Wistar Rats. Neurogastroenterol. Motil. 31, e13523. 10.1111/nmo.13523 30537037

[B72] Trevizan A. R. Vicentino-Vieira S. L. da Silva Watanabe P. Góis M. B. de Melo G. de A. N.. (2016). Kinetics of Acute Infection With *Toxoplasma Gondii* and Histopathological Changes in the Duodenum of Rats. Exp. Parasitol. 165, 22–29. 10.1016/j.exppara.2016.03.015 26993084

[B73] Troncone E. Marafini I. Stolfi C. Monteleone G. (2018). Transforming Growth Factor-β1/Smad7 in Intestinal Immunity, Inflammation, and Cancer. Front. Immunol. 9, 1407. 10.3389/fimmu.2018.01407 29973939 PMC6019438

[B74] Vieira T. da S. Rugani J. N. Nogueira P. M. Torrecilhas A. C. Gontijo C. M. F.. (2019). Intraspecies Polymorphisms in the Lipophosphoglycan of *L. braziliensis* Differentially Modulate Macrophage Activation via TLR4. Front. Cell. Infect. Microbiol. 9, 240. 10.3389/fcimb.2019.00240 31355149 PMC6636203

[B75] Vodovotz Y. Bogdan C. Paik J. Xie Q. W. Nathan C. (1993). Mechanisms of Suppression of Macrophage Nitric Oxide Release by Transforming Growth Factor γ. J. Exp. Med. 178, 605–613. 10.1084/jem.178.2.605 7688028 PMC2191129

[B76] World Health Organization (2020). Global Leishmaniasis Surveillance 2017–2018, and First Report on 5 Additional Indicators (Genebra). Available at: https://apps.who.int/iris/bitstream/handle/10665/339849/WER9608-eng-fre.pdf.

[B77] Yoo B. B. Mazmanian S. K. (2017). The Enteric Network: Interactions Between the Immune and Nervous Systems of the Gut. Immunity 46, 910–926. 10.1016/j.immuni.2017.05.011 28636959 PMC5551410

[B78] Yu Y. Daly D. M. Adam I. J. Kitsanta P. Hill C. J. Wild J.. (2016). Interplay Between Mast Cells, Enterochromaffin Cells, and Sensory Signaling in the Aging Human Bowel. Neurogastroenterol. Motil. 28, 1465–1479. 10.1111/nmo.12842 27206689 PMC5053273

